# The interactions of Bcl9/Bcl9L with β-catenin and Pygopus promote breast cancer growth, invasion, and metastasis

**DOI:** 10.1038/s41388-021-02016-9

**Published:** 2021-09-20

**Authors:** Vida Vafaizadeh, David Buechel, Natalia Rubinstein, Ravi K. R. Kalathur, Lorenzo Bazzani, Meera Saxena, Tomas Valenta, George Hausmann, Claudio Cantù, Konrad Basler, Gerhard Christofori

**Affiliations:** 1 Department of Biomedicine, University of Basel, Basel, Switzerland; 2 Instituto de Biociencias, Biotecnología y Biología Traslacional, Departamento de Fisiología, Biología Molecular y Celular, Facultad de Ciencias Exactas y Naturales, Universidad de Buenos Aires, Buenos Aires, Argentina; 3 Murdoch Children’s Research Institute, Royal Children’s Hospital, Parkville, VIC Australia; 4 Department of Life Sciences, University of Siena, Siena, Italy; 5 Department of Molecular Life Sciences, University of Zürich, Zürich, Switzerland; 6 Wallenberg Centre for Molecular Medicine, Linköping University, Linköping, Sweden; 7 Department of Biomedical and Clinical Sciences, Division of Molecular Medicine and Virology, Faculty of Medicine and Health Sciences, Linköping University, Linköping, Sweden

**Keywords:** Breast cancer, Cancer models, Metastasis, Morphogen signalling

## Abstract

Canonical Wnt/β-catenin signaling is an established regulator of cellular state and its critical contributions to tumor initiation, malignant tumor progression and metastasis formation have been demonstrated in various cancer types. Here, we investigated how the binding of β-catenin to the transcriptional coactivators B-cell CLL/lymphoma 9 (Bcl9) and Bcl9-Like (Bcl9L) affected mammary gland carcinogenesis in the MMTV-PyMT transgenic mouse model of metastatic breast cancer. Conditional knockout of both *Bcl9* and *Bcl9L* resulted into tumor cell death. In contrast, disrupting the interaction of Bcl9/Bcl9L with β-catenin, either by deletion of their HD2 domains or by a point mutation in the N-terminal domain of β-catenin (D164A), diminished primary tumor growth and tumor cell proliferation and reduced tumor cell invasion and lung metastasis. In comparison, the disruption of HD1 domain-mediated binding of Bcl9/Bcl9L to Pygopus had only moderate effects. Interestingly, interfering with the β-catenin-Bcl9/Bcl9L-Pygo chain of adapters only partially impaired the transcriptional response of mammary tumor cells to Wnt3a and TGFβ treatments. Together, the results indicate that Bcl9/Bcl9L modulate but are not critically required for canonical Wnt signaling in its contribution to breast cancer growth and malignant progression, a notion consistent with the “just-right” hypothesis of Wnt-driven tumor progression.

## Introduction

Aberrant canonical Wnt signaling and the resulting dysregulation of β-catenin’s transcriptional functions play key roles in the progression of inflammation, fibrosis, and tumor formation [1, 2]. Canonical TGFβ signaling also plays a key role in the development of these pathological disorders and also efficiently activates an epithelial–mesenchymal transition (EMT) program [3, 4]. EMT is a multistage and central process during malignant tumor progression. It regulates the transitions between epithelial and mesenchymal states and cell plasticity, which contributes to intra-tumoral heterogeneity [5]. The reverse process, a mesenchymal–epithelial transition (MET), appears required for the colonization of metastatic cells in distant organs [6]. In breast cancer, driver mutations in Wnt or in TGFβ signaling pathways are rare, yet downstream effectors and target genes are often induced during malignant tumor progression and metastasis formation [7, 8]. While the functional contribution of TGFβ signaling to EMT, malignant tumor progression, and metastasis formation has been amply demonstrated, the role of Wnt signaling in late-stage breast cancer progression still remains unclear.

β-catenin has two roles: it is a critical component of cadherin-mediated cell–cell adhesion complexes and it is the transcriptional co-activator in canonical Wnt signaling. It interacts with a variety of proteins, including adhesion molecules, cytoplasmic signaling regulators, and nuclear transcriptional regulators [1, 9]. B-cell CLL/lymphoma 9 (Bcl9) and its paralog Bcl9-like (Bcl9L) are among the latter: they bind to the N-terminus of β-catenin and act as auxiliary transcriptional coactivators. Bcl9/Bcl9L simultaneously bind to Pygo and to the N-terminal domain of β-catenin via the HD1 and HD2 domains, respectively [10]. The resulting chain of adapters is required for a maximal output of Wnt/β-catenin pathway in mammalian cells [10–12]. HD1 and HD2 are evolutionarily conserved domains that in mice are required for embryonic development; their individual disruption is lethal [13].

Cantù et al. have generated and studied constitutive knock-in *Bcl9* and *Bcl9L* alleles lacking either the HD1 or the HD2 domains. The work has revealed that Bcl9 and Bcl9L contribute to diverse aspects of development in both β-catenin-dependent and independent manners. For example, they contribute to eye lens development in a HD1-dependent (binding to Pygo2), but HD2-independent (binding to β-catenin) fashion [13]. *Bcl9 and Bcl9L* are direct target genes of Pax6, a master transcriptional regulator of eye development [13, 14]. Moreover, Bcl9 and Bcl9L exert a non-transcriptional cytoplasmic function in enamel formation and hardness by affecting the secretion of enamel-building proteins and iron deposition [15]. The *Bcl9/Bcl9L* mutations also cause cardiac defects in zebrafish and mice by a β-catenin-dependent mechanism, yet the defective embryos still maintained a broad transcriptional activity of β-catenin [16]. Finally, the interaction of Bcl9/Bcl9L with β-catenin is important for the maintenance of intestinal epithelial stem cells by selectively modulating Wnt/β-catenin-mediated transcriptional output [17]. Yet, other transcription factors may also interact with Bcl9/Bcl9L and β-catenin to modulate their transcriptional output, as for example shown for the Tbx3-mediated promotion of colorectal cancer cell metastasis [18].

Enhanced Wnt signaling regulates the maintenance of a stemness state in normal and tumorigenic tissues. For example, knockout of *Bcl9/Bcl9L* in a mouse model of colorectal cancer has resulted in the reprogramming of cancer cells from a stemness state to differentiation manifested by the downregulation of EMT-related gene expression [19]. Importantly, the gene signature of *Bcl9/Bcl9L* knockout cells is negatively associated with the high stemness subtypes of colorectal cancers and positively correlates with patient overall survival [20]. In breast cancer, BCL9 has been identified as a prognostic biomarker for high-risk human ductal carcinoma in situ (DCIS) [21]. Subsequently, BCL9 has been found to form a complex with STAT3 and to enhance the expression of the genes encoding for integrin β3 and its associated metalloproteinase MMP16 [22]. Another study has shown that nuclear BCL9L is associated with high nuclear grade and the expression of ErbB2/HER-2 in both DCIS and invasive ductal carcinoma (IDC) [23]. How BCL9 and BCL9L contribute to the breast cancer progression is not clear.

Here, we have analyzed the effect of interfering with the Bcl9/Bcl9L branch of Wnt signaling on tumor cell growth and invasion in a mouse model of metastatic breast cancer (FVB/N-Tg(MMTV-PyVT)634Mul/J) [24]. MMTV-PyMT transgenic mice form multifocal tumors in mammary glands which progress from hyperplasia, adenoma to invasive carcinoma and finally seed metastases to the lungs, thus recapitulating the luminal B subtype of human breast cancer with ErbB2 overexpression [25]. Cell lines derived from tumors of these mice offer suitable models for TGFβ-induced EMT in vitro and in vivo [26, 27], and the blockade of TGFβ has inhibited tumor cell viability, migration, and lung metastases [28]. We have found that the disruption of the interaction of β-catenin with Bcl9/Bcl9L proteins in tumor cells of MMTV-PyMT transgenic mice causes a significant reduction in primary tumor growth and metastasis formation. Tumor cell proliferation is reduced, tumor cell differentiation is increased and EMT is prevented. The results suggest that in breast cancer Bcl9/9L-dependent Wnt/β-catenin signaling regulates genes promoting tumor cell growth and metastasis.

## Results

### Wnt/β-catenin signaling is activated during TGFβ-induced EMT

We have previously induced EMT and MET in the Py2T and 1099-PyMT murine breast cancer cell lines derived from tumors of MMTV-PyMT transgenic mice and performed RNA-sequencing [26, 27, 29]. Differential gene expression analysis revealed that expression of many components of the Wnt signaling pathway substantially changed during TGFβ-induced EMT and TGFβ withdrawal-induced MET (Supplementary Fig. 1A). Py2T cells gradually transitioned from epithelial to mesenchymal cell morphology during 10 days of TGFβ treatment (Supplementary Fig. 1B). This phenotype change correlated with the loss of the expression of the epithelial cell adhesion molecule E-cadherin and an increased expression of mesenchymal markers, such as N-cadherin and fibronectin (Supplementary Fig. 1C). With the induction of EMT biomarkers, the expression of Bcl9, integrin α5 (Itga5), and β-catenin target genes, such as Axin2, Lef1, and Wls, was increased (Supplementary Fig. 1C, D). However, repeated experiments revealed that canonical Wnt signaling, as determined by immunoblotting for non-phosphorylated (activated) β-catenin, appeared to be activated already in epithelial cells. It is then found reduced during the early stages of EMT and to re-appear at the later stages of EMT. Total β-catenin was rather decreased during EMT most likely due to the dissolutions of adherens junctions during EMT and the subsequent degradation of some of β-catenin (Supplementary Fig. 1C).

**Fig. 1:**
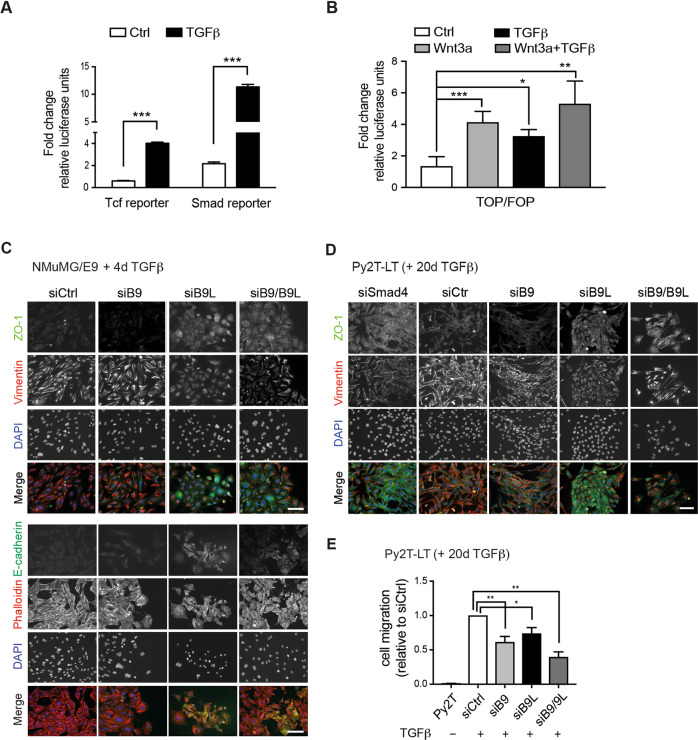
Canonical Bcl9/Bcl9L-dependent Wnt signaling during TGFβ-induced EMT. **A** Tcf and Smad-promoter luciferase reporter assays (Qiagen *Cignal Lenti* System) were used to monitor canonical Wnt-mediated and canonical TGFβ transcriptional outputs during TGFβ-induced EMT of Py2T cells. The graph represents luciferase activity units of Tcf and Smad reporters relative to negative controls Py2T cells 7 days after TGFβ treatment. *Firefly* luciferase activity measurements were normalized to *Renilla* luciferase activity. Data are presented as mean ± SEM. Statistical analysis was performed using the unpaired *t*-test. ***p < 0.001. **B** TOPflash (TCL/LEF-Firefly luciferase) and FOPflash (Neg control, mutated Tcf-Lef-binding site) luciferase reporter assay in Py2T cells treated with Wnt3a or TGFβ or both. *Firefly* luciferase activity measurements were normalized to *Renilla* luciferase activity. Data are presented as mean ± SEM. Statistical analysis was performed using the ordinary one-way ANOVA multiple comparison test. *p < 0.05; **p < 0.01; ***p < 0.001. **C** siRNA-mediated depletion of Bcl9 and Bcl9L prevents TGFβ-induced EMT. NMuMG/E9 cells were transfected with control siRNA (siCtrl) or siRNAs against *Bcl9* (siB9), *Bcl9L* (siB9L) or both (siB9/B9L) 2 days before starting the TGFβ treatment for further 4 days. Immunofluorescence microscopy analysis visualized the epithelial markers tight junction protein-1 (ZO-1) and E-cadherin and the mesenchymal marker Vimentin. Fluorescently labeled phalloidin visualized the actin cytoskeleton, and nuclei were counterstained with DAPI. Scale bar, 100 µm. **D** siRNA-mediated depletion of Bcl9 and Bcl9L partially reverses TGFβ-induced EMT. Mesenchymal Py2T cells previously treated for >20 days (Py2T-LT) were transfected with control siRNA (siCtrl) or siRNAs against *Bcl9* (siB9), *Bcl9L* (siB9L), or both (siB9/B9L). Immunofluorescence microscopy analysis visualized the epithelial markers tight junction protein-1 (ZO-1) and the mesenchymal marker Vimentin. Nuclei were counterstained with DAPI. Scale bar, 100 µm. **E** siRNA-mediated depletion of Bcl9 and Bcl9L reduces migration of Py2T-LT cells. Untreated epithelial Py2T cells or mesenchymal Py2T cells previously treated for >20 days (Py2T-LT) were transfected with siCtrl, siB9, siB9L or both (siB9/B9L) and allowed to migrate for 18 h along a serum gradient in a Transwell migration assay. Migrated cells were stained with DAPI and quantified relative to siCtrl-transfected cells. Data are presented as mean ± SEM. Statistical analysis was performed using ordinary one-way ANOVA multiple comparison test. *p < 0.05; **p < 0.01.

Next, we assessed whether β-catenin/Tcf transcriptional activity was increased during TGFβ-induced EMT using a Tcf motif-containing luciferase reporter construct. A construct containing Smad-binding motifs was used as a control of effective EMT induction. Treatment of Py2T cells with TGFβ for 7 days significantly increased both Smad-mediated and β-catenin/Tcf-mediated transcriptional activities (Fig. 1A). In addition, in Py2T cells transfected with the TCF reporter plasmid TOPFlash treatment with TGFβ or the canonical Wnt ligand Wnt3a comparably induced β-catenin’s transcriptional activity (Fig. 1B).

These results raise the possibility that canonical Wnt signaling may contribute to TGFβ-induced EMT in murine breast cancer cells.

### Bcl9 and Bcl9L contribute to TGFβ-induced EMT in vitro

To test the role of Wnt signaling in TGFβ-induced EMT, we ablated the expression of Bcl9 and Bcl9L alone (siB9 and siB9L) or together (siB9/B9L) during TGFβ-induced EMT in non-tumorigenic normal murine mammary gland epithelial cells (NMuMG/E9) and in tumorigenic Py2T cells [26, 29, 30]. Cells were transfected with control siRNA (siCtrl), siB9, siB9L or siB9/B9L 2 days before TGFβ treatment for 4 days to monitor potential effects on EMT initiation. siRNA-mediated knockdown efficiently reduced the levels of Bcl9 and Bcl9L proteins (Supplementary Fig. 2A). Morphogenic changes during EMT were assessed by immunofluorescence microscopy analysis of the expression and localization of epithelial and mesenchymal markers. Regardless of the levels of Bcl9 and Bcl9L, in the absence of TGFβ treatment the cells retained their epithelial phenotype (Supplementary Fig. 2B). In contrast, treatment with TGFβ initiated EMT in siCtrl-transfected NMuMG cells: the tight junction protein Zonula occludens-1 (ZO-1) and the adherens junction protein E-cadherin were down-regulated, mesenchymal markers, such as vimentin, were upregulated and actin stress fibers formed, as determined by phalloidin staining (Fig. 1C). Compared to siCTrl-transfected cells, transfection with siB9/B9L and to a lesser extent with siB9L inhibited TGFβ-induced EMT: these cells still formed epithelial cell clusters and expressed ZO-1 and E-cadherin at their cell membranes (Fig. 1C). Knockdown of Bcl9 alone was not sufficient to substantially affect the induction of EMT, although the protein was substantially depleted (Supplementary Fig. 2A).

To study the functional contribution of Bcl9 and Bcl9L to the maintenance of an EMT process, Py2T cells were treated for >20 days with TGFβ to undergo a full EMT (Py2T-LT) and then transfected with siCtrl, siB9, and siB9L. Cells transfected with siB9L alone or in combination with siB9 partially returned to an epithelial morphology and re-expressed ZO-1 at the cell membranes to form epithelial cell clusters (Fig. 1D).

Since EMT is strongly implicated in cancer cell migration [3], we tested the effects of B9/B9L knockdown on the ability of Py2T cells pretreated with TGFβ for 20 days (Py2T-LT) to migrate toward a serum gradient using a Transwell migration assay. Knockdown of siB9 and siB9L, and most efficiently the combination of both, caused a significant reduction of Py2T cell migration (Fig. 1E).

In conclusion, the results show that Bcl9 and more prominently Bcl9L contribute to the initiation and maintenance of TGFβ-induced EMT and cell migration of cultured mammary tumor cells in vitro, but that they are not exclusive determinants of these TGFβ-induced processes.

### Bcl9 and Bcl9L are highly expressed in invasive carcinomas

**Fig. 2:**
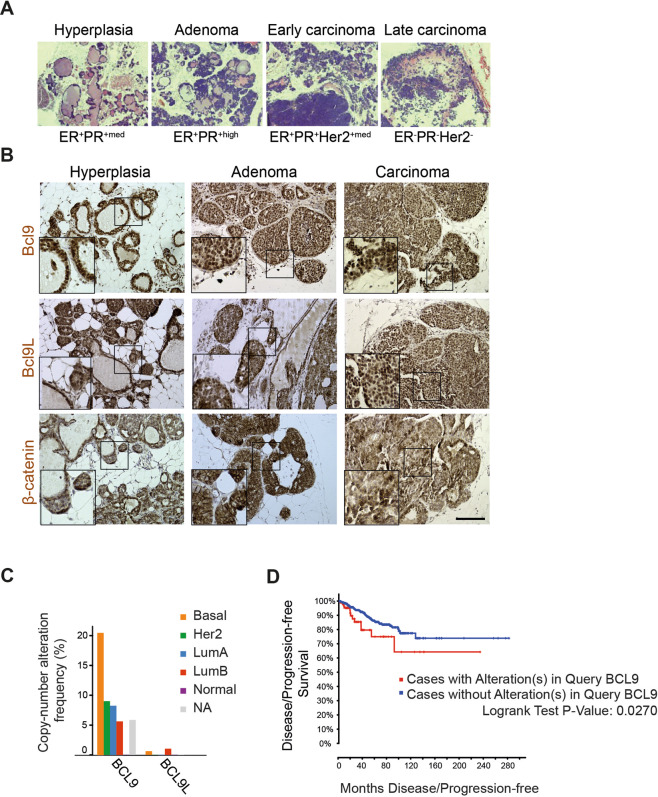
Bcl9 and Bcl9L are highly expressed in invasive breast cancers. **A** Malignant tumor progression in MMTV-PyMT transgenic mice. Histological sections taken from tumors of MMTV-PyMT transgenic mice were stained with hematoxylin and eosin to visualize the different cancer progression stages (hyperplasia: increased amount of single cell layer epithelial tissue: adenoma: increased volumes of multi-layered epithelial structures; early invasive ductal carcinoma: invasion of tumor fronts, loss of epithelial differentiation; late invasive ductal carcinoma: single cell invasion, substantial loss of epithelial differentiation). The expression of estrogen receptor (ER), progesterone receptor (PR), and Her2 in the different progression stages is indicated below the panels. **B** Bcl9, Bcl9L, and β-catenin protein expression during MMTV-PyMT tumor progression. Histological sections taken from tumors of MMTV-PyMT transgenic mice were stained for Bcl9, Bcl9L, and β-catenin protein in different stages of MMTV-PyMT tumor progression. Rectangles indicate higher magnifications to visualize nuclear localization of Bcl9, Bcl9L, and β-catenin. Scale bar, 100 μm. **C** Analysis of *BCL9* and *BCL9L* copy number alterations frequencies in different human breast cancer (BRCA) subtypes reveals a high enrichment of *BCL9* amplifications in basal, Her2, luminal A and B breast cancer subtypes and, in comparison, a lower enrichment of *BCL9L* amplifications in the luminal B subtype. NA not annotated. **D** Disease/progression-free Kaplan–Meier (disease free status since initial treatment) estimate for patients with and without *BCL9* gene alterations (cBioportal online tool). In contrast to *BCL9, BCL9L* gene alterations showed no significant impact on clinical outcome in this data set (not shown).

Previous studies have shown that Bcl9 and Bcl9L are expressed during the different stages of mouse mammary gland development, with a reduction of Bcl9L expression during post-lactational involution [31]. Motivated by this and the knowledge of a functional requirement of Bcl9 and Bcl9L for EMT in MMVT-PyMT-derived tumor cell lines in vitro, we next studied the expression of both proteins during the different stages of tumor progression in mammary glands of MMTV-PyMT transgenic mice in vivo. MMTV-PyMT mammary tumors start to form upon activation of the MMTV promoter during puberty. Tumors progress from hyperplasia to adenoma and finally to an IDC phenotype. This progression correlates with a reduced expression of hormone receptors in the advanced carcinoma stages and with an increase of lung metastasis at the age of 12–14 weeks (Fig. 2A) [24]. Immunohistochemical staining for Bcl9, Bcl9L, and β-catenin protein on paraffin sections from primary tumors of MMTV-PyMT revealed that cytoplasmic and nuclear Bcl9 and Bcl9L and nuclear β-catenin increased during the progression to IDC (Fig. 2B).

Murine and human *Bcl9* and *Bcl9L* proteins are highly conserved (Supplementary Fig. 2C). To examine the relevance of Bcl9 and Bcl9L in tumor progression and invasion in breast cancer patients, we first analyzed the genetic alteration of both genes in breast cancer patient data (from TCGA-BRCA PanCancer Atlas data collection). These data showed a frequency of gene alterations of 11% for *BCL9* and of 1.3% for *BCL9L*, with a frequency of 9.13% and 0.28% for gene amplification, respectively (Supplementary Fig. 3A). Heatmap analysis indicated a high expression of *BCL9* and *BCL9L* in patients with any kind of genetic alterations in their *BCL9* and *BCL9L* genes (cBioportal online tool) (Supplementary Fig. 3A). The frequency of *BCL9L* gene amplification in different breast cancer subtypes is highest in basal and luminal B breast cancer subtypes, with less incidence in the other subtypes and none in normal-type breast cancer (Fig. 2C). *BCL9* amplification correlated with poor prognosis in breast cancer patients (cBioportal online tool) (Fig. 2D). Consistent with our analysis, using a separate breast cancer data set (provisional TCGA; 959 cases) Elsarraj et al. reported significant levels of gene alteration for *BCL9* (26%) and *BCL9L* (5%) in invasive breast cancers, mainly through gene amplification and partly by mRNA upregulation [21]. Using the same TCGA breast cancer data set we also found that low expression levels of *BCL9L* gene were a prognostic marker in ER^-^PR^-^ patients (Supplementary Fig. 3B–D). In contrast, the high gene amplification and expression of *BCL9* observed in this patient data set did not reveal a significant correlation with disease outcome in any of the breast cancer subtypes (Supplementary Fig. 3B–D).

These results show that Bcl9/Bcl9L proteins are expressed and potentially deregulated in invasive carcinoma in the MMTV-PyMT mouse model and in breast cancer patients.

### Complete loss of Bcl9 and Bcl9L functions affects tumor cell survival

**Fig. 3:**
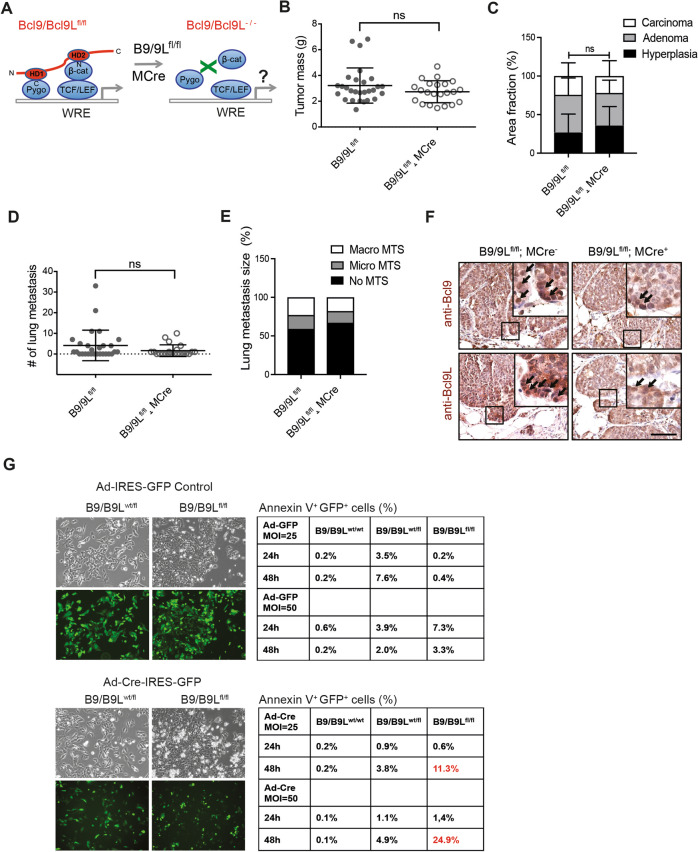
Loss of both *Bcl9* and *Bcl9L* gene functions affects tumor cell survival. **A** Schematic representation of the knockout strategy leading to the complete loss of Bcl9 and Bcl9L in MMTV-PyMT mammary tumors. Mice carrying floxed alleles of Bcl9 and Bcl9L (B9/B9L^fl/fl^) were crossed with MMTV-PyMT and MMTV-Cre mice to generate composite transgenic mice lacking the expression of Bcl9 and Bcl9L in mammary tumor cells (B9/B9L^−/−^). Bcl9 and Bcl9L act as coactivators in β-catenin-mediated transcription and directly bind to the N-terminus of β-catenin through their HD2 domains and to the C-terminus of Pygopus (Pygo) through their HD1 domains. **B** Tumor growth in B9/B9L^fl/fl^ transgenic mice (wild-type; *n* = 22) and B9/B9L^fl/fl^;MCre transgenic mice (tumor cell-specific knockout; *n* = 28). Tumor mass from 12-week-old females was calculated as total weight of thoracic, abdominal, and inguinal mammary glands with tumors lesions. Each dot represents one mouse. Data are presented as mean ± SEM. Statistical analysis was performed using unpaired *t*-test. **C** The bar graph represents the effects of tumor cell-specific knockout of B9/B9L on the differentiation stage of primary tumors. Quantification of tumor stages (hyperplasia, adenoma, and carcinoma) are shown for primary tumors based on histological analysis of stitched microscopy images of different tumor areas. Data are presented as mean ± SEM. Statistical analysis was performed using ordinary one-way ANOVA multiple comparison test. **D** Lung metastasis in B9/B9L^fl/fl^ transgenic mice (wild-type; *n* = 22) and B9/B9L^fl/fl^;MCre transgenic mice (tumor cell-specific knockout; *n* = 28). Lung metastasis nodules were counted in paraffin-embedded lung tissues, which were serially sectioned and stained with H&E. Each dot represents the total number of lung metastases per mouse. Data are presented as mean ± SEM. Statistical analysis was performed using unpaired *t*-test. **E** The bar graph represents the effects of tumor cell-specific knockout of B9/B9L on the outgrowth of lung metastases as no metastasis, micrometastases (<100 clustered cells) and macrometastases (large nodules). The size of lung metastasis was determined in serially sectioned paraffin-embedded lung tissues isolated from the mice described in D. **F** Immunostaining of Bcl9 and Bcl9L proteins on tumor sections of B9/B9L^fl/fl^ and B9/B9L^fl/fl^;MCre transgenic mice. Nuclear expression of Bcl9 and Bcl9L proteins are shown in higher magnifications. Note that despite the expression of Cre recombinase in B9/B9L^fl/fl^;MCre transgenic mice the expression of Bcl9 and Bcl9L proteins was not reduced. Scale bar, 100 µm. **G** Loss of B9/B9L induces tumor cell apoptosis. Three tumor cell lines were established from tumors of MMTV-PyMT B9/B9L^wt/fl^ and B9/B9L^fl/fl^ mice. The cell lines were then infected with adenovirus expressing only GFP (Ad-IRES-GFP control) or adenovirus expressing Cre recombinase and GFP (Ad-Cre-IRES-GFP). Two different multiplicity of infection (MOI = 25 and 50) and analysis at 24 and 48 h after infection were performed. Cells infected with adenovirus and expressing GFP are shown by phase contrast and immunofluorescence microscopy pictures at the left. Cells were stained with Annexin V, and Annexin V^+^GFP^+^ apoptotic cells were quantified by flow cytometry, demonstrating that B9/B9L are required for cell survival, as shown on the right.

To investigate the functional relevance of Bcl9 and Bcl9L during tumor progression and invasion, we genetically deleted both *Bcl9* and *Bcl9L* genes in mammary tumor cells of MMTV-PyMT mice. The simultaneous targeting of both proteins was chosen to avoid any compensatory effect. MMTV-PyMT mice were crossed with Bcl9^fl/fl^ and Bcl9L^fl/fl^ mice [19] and with MMTV-Cre mice, expressing Cre-recombinase exclusively in mammary epithelial cells [32]. Bcl9^fl/fl^;Bcl9L^fl/fl^;MMTV-PyMT with and without MMTV-Cre expression were designated B9/B9L^fl/fl^ and B9/B9L^fl/fl^;MCre genotype mice, respectively (Fig. 3A). Surprisingly, at the age of 12 weeks, tumor growth (tumor mass) and the number of lung metastases were similar in B9/B9L^fl/fl^;MCre mice and B9/B9L^fl/fl^ control mice (Fig. 3B). There were also no significant differences in the percentage of hyperplasia, adenoma, and carcinoma areas and in the number and size of lung metastases between B9/B9L^fl/fl^ control and B9/B9L^fl/fl^;MCre mice (Fig. 3C–E). The reason seems to be a failure to delete both *Bcl9* and *Bcl9L* genes in these tumors (Supplementary Fig. 3E). Immunohistochemical staining also revealed that Bcl9 and Bcl9L proteins were both still expressed in cytosols and nuclei of tumor cells of MCre^+^ genotype (Fig. 3F), suggesting either a low recombination efficiency or a competitive selection against recombined tumor cells.

To confirm the functionality of Cre recombinase expressed by MMTV-Cre, B9/B9L^wt/wt^;Mpy;MCre and B9/B9L^fl/fl^;Mpy;MCre mice were crossed with GFP-reporter mice (R26-LSL-GFP). At 5 weeks of age, when Cre is first expressed in mammary epithelial cells, the amount of GFP was comparable between B9/B9L^fl/fl^;MCre mice and B9/B9L^wt/wt^;MCre mice (Supplementary Fig. 3F, G). Interestingly, at 12 weeks of age the percentage of GFP-positive cells was strongly reduced in B9/B9L^fl/fl^;Mpy;MCre mice (Supplementary Fig. 3F, G). These results suggest there is a selection against tumor cells lacking both Bcl9 and Bcl9L.

To further validate whether there was a selection against tumor cells lacking both Bcl9 and Bcl9L, we examined whether the genetic deletion of *Bcl9/Bcl9L* affected tumor cell survival. We established cell lines from tumors of B9/B9L^wt/wt^, B9/B9L^wt/fl^, and B9/B9L^fl/fl^ genotypes and infected these cells with Adenoviruses expressing either only GFP (Ad-IRES-GFP) or expressing both GFP and Cre-recombinase (Ad-Cre-IRES-GFP) at a multiplicity of infection of 25 or 50. After 24 or 48 h, the percentage of Annexin V^+^GFP^+^ apoptotic cells was measured by flow cytometry. Consistent with the hypothesis counterselection, expression of Cre-recombinase in B9/B9L^fl/fl^ cells induced apoptosis, in particular after 48 h of viral infection (Fig. 3G).

Together these results indicate that the complete loss of Bcl9 and Bcl9L function in mammary tumor cells provokes their apoptotic death. As a consequence, there is a selection against Bcl9/Bcl9L-deficient cells. Such a lethal effect upon complete loss of Bcl9 and Bcl9L expression has also been reported during embryonic development [13].

### The interactions of Bcl9/Bcl9L with Pygopus moderately contribute to tumor progression

As shown above, Bcl9/Bcl9L contribute to the initiation and maintenance of TGFβ-induced EMT and cell migration in vitro, yet the complete loss of Bcl9/Bcl9L has led to tumor cell apoptosis in vitro and in vivo. We thus were motivated to investigate the specific contribution of Bcl9 and Bcl9L to β-catenin-mediated Wnt signaling and as a consequence to tumor progression in the MMTV-PyMT transgenic mouse model of breast cancer. Toward this end, we first used knock-in mouse lines carrying in-frame deletions of the conserved HD1 (Bcl9/Bcl9L^ΔHD1/wt^) [13]. This deletion ablates the interactions of Bcl9 and Bcl9L with Pygopus. Since B9/B9L double-homozygous mutants of HD1 were lethal [13], we crossed heterozygous Bcl9/Bcl9L^ΔHD1/fl^ mutant mice with MMTV-PyMT transgenic mice to induce mammary tumor formation and with MMTV-Cre mice to delete the floxed allele in mammary tumors of Bcl9/Bcl9L^ΔHD1/fl^ mutant mice.

**Fig. 4:**
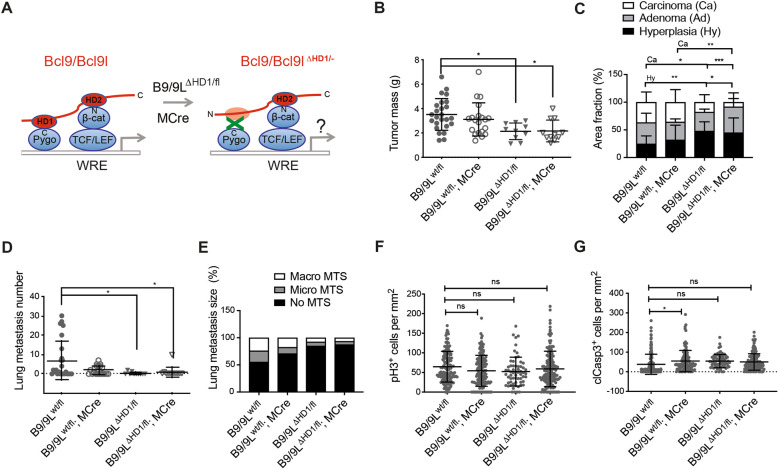
Disrupting the interaction of B9/B9L with Pygopus moderately reduces primary tumor growth and malignant tumor progression. **A** Schematic representation of the knock-in strategy to express the ΔHD1 mutant forms of Bcl9 and Bcl9L and thus disrupting the interaction of Bcl9 and Bcl9L with Pygopus in mammary tumor cells of MMTV-PyMT transgenic mice. Mice carrying floxed alleles of Bcl9 and Bcl9L and carrying the knock-in ΔHD1 mutant alleles of Bcl9 and Bcl9L were crossed with MMTV-PyMT and MMTV-Cre mice to generate composite transgenic mice expressing exclusively the ΔHD1 mutant forms of Bcl9 and Bcl9L in mammary tumor cells (Bcl9/Bcl9L^ΔHD1/fl^;MMTV-PyMT;MMTV-Cre). **B** Tumor growth in heterozygous B9/B9L^ΔHD1/fl^ transgenic mice with (*n* = 12) or without MCre (*n* = 10) compared to control B9/B9L^wt/fl^ mice with (*n* = 20) or without MCre (*n* = 25) expression. Tumor mass from 12-week-old females was calculated as total weight of thoracic, abdominal, and inguinal mammary glands with tumors lesions. Each dot represents one mouse. Data are presented as mean ± SEM. Statistical analysis was performed using ordinary one-way ANOVA multiple comparison test. *p < 0.05. **C** The bar graph represents the effects of tumor cell-specific expression of the ΔHD1 mutant forms of Bcl9 and Bcl9L on malignant tumor progression of primary tumors in B9/B9L^ΔHD1/fl^;MMTV-PyMT;MMTV-Cre transgenic mice. Quantification of tumor stages (hyperplasia, adenoma, and carcinoma) are shown for primary tumors based on histological analysis of stitched microscopy images of different tumor areas. Data are presented as mean ± SEM. Statistical analysis was performed using ordinary one-way ANOVA multiple comparison test. *p < 0.05; **p < 0.01; ***p < 0.001. **D** Number of lung metastasis in heterozygous B9/B9L^ΔHD1/fl^ transgenic mice with (*n* = 12) or without MCre (*n* = 10) compared to control B9/B9L^wt/fl^ mice with (*n* = 20) or without MCre (*n* = 25) expression. Lung metastasis nodules were counted in paraffin-embedded lung tissues, which were serially sectioned and stained with H&E. Each dot represents the total number of lung metastases per mouse. Data are presented as mean ± SEM. Statistical analysis was performed using ordinary one-way ANOVA multiple comparison test. *p < 0.05. **E** The bar graph represents the effects of tumor cell-specific expression of the ΔHD1 mutant forms of Bcl9 and Bcl9L on the outgrowth of lung metastases. The size of lung metastasis was determined in serially sectioned paraffin-embedded lung tissues isolated from the mice described in D. **F** Quantification of immunostaining for the mitosis marker phospho-histone 3 (pH3) on tumor sections of B9/B9L^ΔHD1/fl^ transgenic mice with or without MCre compared to control B9/B9L^wt/fl^ mice with or without MCre. Each data point represents one histological section. Data are presented as mean ± SEM. Statistical analysis was performed using ordinary one-way ANOVA multiple comparison test. ns not significant. **G** Quantification of immunostaining for the apoptosis marker cleaved Caspase-3 (clCasp3) on tumor sections of B9/B9L^ΔHD1/fl^ transgenic mice with or without MCre compared to control B9/B9L^wt/fl^ mice with or without MCre. Each data point represents one histological section. Data are presented as mean ± SEM. Statistical analysis was performed using ordinary one-way ANOVA multiple comparison test. *p < 0.05; ns not significant.

In Bcl9/Bcl9L^ΔHD1/fl^;MMTV-PyMT;MMTV-Cre mice (B9/B9L^ΔHD1/fl^;MCre), interaction of B9/B9L with Pygopus was disrupted (Fig. 4A). At 12 weeks of age of this mice, primary tumor growth was significantly reduced (Fig. 4B). Disruption of the B9/B9L-Pygopus interactions also caused a reduction of tumor progression and invasive phenotypes in primary tumors (Fig. 4C). Likewise we also observed a reduction in the numbers of lung metastasis and their nodular outgrowth (Fig. 4D, E). A comparable reduction in tumor progression was observed in B9/B9L^ΔHD1/fl^ mice without MCre, yet not in B9/B9L^wt/fl^ mice with MCre. This suggests that the B9/B9L-ΔHD1 mutant proteins exerted a dominant-negative effect over wild-type B9/B9L and that there was no haploinsufficient effect caused by the ablation of single alleles of Bcl9 and Bcl9L.

To delineate the basis for the repressive effect of B9/B9L-ΔHD1 on primary tumor growth, we monitored potential changes in tumor cell proliferation and in tumor cell apoptosis by quantification of immunofluorescence staining for the mitosis marker phospho-histone 3 (PH3) and for the early apoptosis marker cleaved Caspase-3 (clCasp3), respectively (Supplementary Fig. 4A, B). These analyses did not show any significant differences in the rates of tumor cell proliferation or of apoptosis in B9/B9L-ΔHD1 tumors compared to wild-type controls (Fig. 4F, G). This might explain why the effect of the ΔHD1 mutations were relatively moderate on primary tumor growth and metastasis formation. In summary the interaction of Bcl9/Bcl9L with Pygopus appears to play only a limited role in the regulation of breast cancer tumor progression.

### The interactions of Bcl9/Bcl9L with β-catenin substantially contribute to tumor progression

**Fig. 5:**
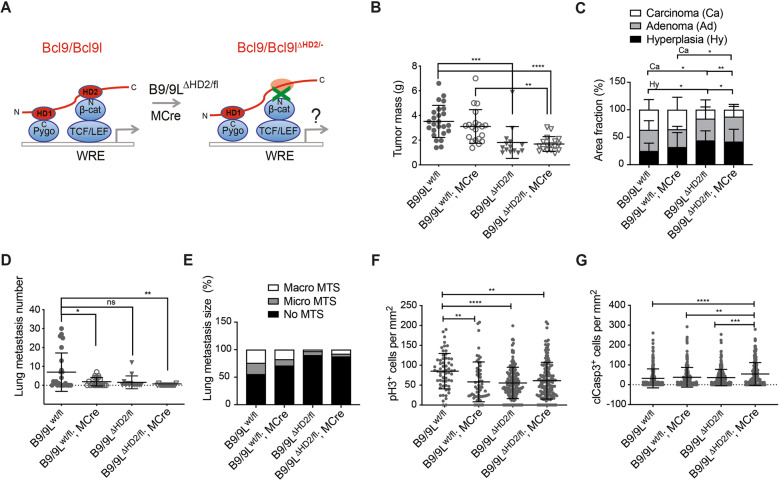
Disrupting the interaction of B9/B9L with β-catenin substantially reduces primary tumor growth and malignant tumor progression and metastasis. **A** Schematic representation of the knock-in strategy to express the ΔHD2 mutant forms of Bcl9 and Bcl9L and thus disrupting the interaction of Bcl9 and Bcl9L with β-catenin in mammary tumor cells of MMTV-PyMT transgenic mice. Mice carrying floxed alleles of Bcl9 and Bcl9L and carrying the knockin ΔHD2 mutant alleles of Bcl9 and Bcl9L were crossed with MMTV-PyMT and MMTV-Cre mice to generate composite transgenic mice expressing exclusively the ΔHD2 mutant forms of Bcl9 and Bcl9L in mammary tumor cells (B9/B9L^ΔHD2/fl^;MMTV-PyMT;MMTV-Cre). **B** Tumor growth in heterozygous B9/B9L^ΔHD2/fl^ transgenic mice with (*n* = 16) or without MCre (*n* = 14) compared to controls B9/B9L^wt/fl^ mice with (*n* = 20) or without MCre (*n* = 25) expression. Tumor mass from 12-week-old females was calculated as total weight of thoracic, abdominal, and inguinal mammary glands with tumors lesions. Each dot represents one mouse. Data are presented as mean ± SEM. Statistical analysis was performed using ordinary one-way ANOVA multiple comparison test. **p < 0.01; ***p < 0.001; ****p < 0.0005. **C** The bar graph represents the effects of tumor cell-specific expression of the ΔHD2 mutant forms of Bcl9 and Bcl9L on malignant tumor progression of primary tumors in Bcl9/Bcl9L^ΔHD2/fl^;MMTV-PyMT;MMTV-Cre transgenic mice. Quantification of tumor stages (hyperplasia, adenoma, and carcinoma) are shown for primary tumors based on histological analysis of stitched microscopy images of different tumor areas. Data are presented as mean ± SEM. Statistical analysis was performed using ordinary one-way ANOVA multiple comparison test. *p < 0.05; **p < 0.01. **D** Number of lung metastasis in heterozygous B9/B9L^ΔHD2/fl^ transgenic mice with (*n* = 16) or without MCre (*n* = 14) compared to controls B9/B9L^wt/fl^ mice with (*n* = 20) or without MCre (*n* = 25) expression. Lung metastasis nodules were counted in paraffin-embedded lung tissues, which were serially sectioned and stained with H&E. Each dot represents the total number of lung metastases per mouse. Data are presented as mean ± SEM. Statistical analysis was performed using ordinary one-way ANOVA multiple comparison test. *p < 0.05; **p < 0.01; ns not significant. **E** The bar graph represents the effects of tumor cell-specific expression of the ΔHD2 mutant forms of Bcl9 and Bcl9L on the outgrowth of lung metastases. The size of lung metastasis was determined in serially sectioned paraffin-embedded lung tissues isolated from the mice described in D. **F** Quantification of immunostaining for the mitosis marker phospho-histone 3 (pH3) on tumor sections of B9/B9L^ΔHD2/fl^ transgenic mice with or without MCre compared to control B9/B9L^wt/fl^ mice with or without MCre. Each data point represents one histological section. Data are presented as mean ± SEM. Statistical analysis was performed using ordinary one-way ANOVA multiple comparison test. **p < 0.01; ****p < 0.0005. **G** Quantification of immunostaining for the apoptosis marker cleaved Caspase-3 (clCasp3) on tumor sections of B9/B9L^ΔHD2/fl^ transgenic mice with or without MCre compared to control B9/B9L^wt/fl^ mice with or without MCre. Each data point represents one histological section. Data are presented as mean ± SEM. Statistical analysis was performed using ordinary one-way ANOVA multiple comparison test. **p < 0.01; ***p < 0.001; ****p < 0.0005.

We next ablated the specific interaction of Bcl9 and Bcl9L with β-catenin by using knock-in mouse lines carrying an in-frame deletion of their conserved HD2 domains [13]. Since B9/B9L double-homozygous mutants of HD2 were lethal [13], we crossed heterozygous Bcl9/Bcl9L^ΔHD2/fl^ mutant mice with MMTV-PyMT transgenic mice to induce mammary tumor formation and with MMTV-Cre mice to delete the floxed alleles in mammary tumors of Bcl9/Bcl9L^ΔHD2/fl^ mutant mice (Fig. 5A). We then assessed how disrupting the interaction of B9/B9L with β-catenin affected primary tumor growth, tumor progression, and metastasis formation. At 12 weeks of age, primary tumor growth, tumor progression to invasive cancer and the numbers and sizes of lung metastases were significantly reduced in B9/B9L^ΔHD2/fl^;MCre mice (Fig. 5B–E). Similar to the B9/B9L-ΔHD1, B9/B9L-ΔHD2 seemed be dominant over wild-type B9/B9L: tumor growth and progression were comparable between B9/B9L^ΔHD2/fl^ mice and B9/B9L^ΔHD2/fl^;MCre mice, while B9/B9L^wt/fl^;MCre mice did not show any difference to B9/B9L^wt/fl^ mice (Fig. 5B–E), again excluding a haploinsufficient effect. Immunofluorescence microscopy analysis of tumor sections stained with PH3 or clCasp3 revealed that the reduced tumor growth in B9/B9L-ΔHD2-expressing mice was due to both diminished tumor cell proliferation and increased apoptosis (Fig. 5F, G and Supplementary Fig. 4A, B).

**Fig. 6:**
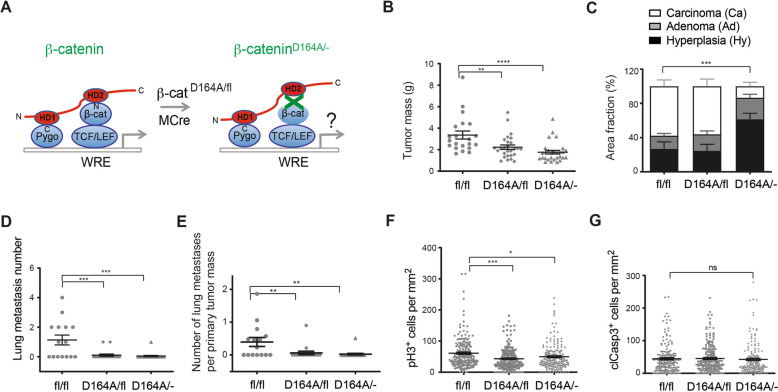
Disrupting the interaction of β-catenin with B9/B9L substantially reduces primary tumor growth and malignant tumor progression and metastasis. **A** Schematic representation of the knock-in strategy to express the D164A mutant form of β-catenin and thus disrupting the interaction of β-catenin with Bcl9 and Bcl9L in mammary tumor cells of MMTV-PyMT transgenic mice. Mice carrying floxed alleles of β-catenin and carrying the knockin D164A mutant allele of β-catenin were crossed with MMTV-PyMT and MMTV-Cre mice to generate composite transgenic mice expressing exclusively the ΔHD2 mutant forms of Bcl9 and Bcl9L in mammary tumor cells (βcatenin^D164A/fl^;MMTV-PyMT;MMTV-Cre). **B** Tumor growth in heterozygous β-catenin^D164A/fl^ transgenic mice with Cre (D164A/-; *n* = 29) or without Cre (D164A/fl; *n* = 26) compared to control β-catenin^fl/fl^ mice without Cre expression (fl/fl; *n* = 21). Tumor mass from 12-week-old females was calculated as total weight of thoracic, abdominal, and inguinal mammary glands with tumors lesions. Each dot represents one mouse. Data are presented as mean ± SEM. Statistical analysis was performed using ordinary one-way ANOVA multiple comparison test. **p < 0.01; ****p < 0.0005. **C** The bar graph represents the effects of tumor cell-specific expression of the D164A mutant form of β-catenin on malignant tumor progression of primary tumors in MMTV-PyMT transgenic mice. Quantification of tumor stages (hyperplasia, adenoma, and carcinoma) are shown for primary tumors based on histological analysis of stitched microscopy images of different tumor areas. Data are presented as mean ± SEM. Statistical analysis was performed using ordinary one-way ANOVA multiple comparison test. ***p < 0.001. **D** Number of lung metastasis in heterozygous β-catenin^D164A/fl^ transgenic mice with Cre (D164A/-; *n* = 19) or without Cre (D164A/fl; *n* = 18) compared to control β-catenin^fl/fl^ mice without Cre expression (fl/fl; *n* = 15). Lung metastasis nodules were counted in paraffin-embedded lung tissues, which were serially sectioned and stained with H&E. Each dot represents the total number of lung metastases per mouse. Data are presented as mean ± SEM. Statistical analysis was performed using ordinary one-way ANOVA multiple comparison test. ***p < 0.001. **E** Relation of the number of lung metastases per primary tumor mass (metastatic index) as taken from the data from B and D. Data are presented as mean ± SEM. Statistical analysis was performed using ordinary one-way ANOVA multiple comparison test. **p < 0.01. **F** Quantification of immunostaining for the mitosis marker phospho-histone 3 (pH3) on tumor sections of β-catenin^D164A/fl^ transgenic mice with Cre (D164A/-) or without Cre (D164A/fl) compared to control β-catenin^fl/fl^ mice without Cre expression (fl/fl). Each data point represents one histological section. Data are presented as mean ± SEM. Statistical analysis was performed using ordinary one-way ANOVA multiple comparison test. *p < 0.05; ***p < 0.001. **G** Quantification of immunostaining for the apoptosis marker cleaved Caspase-3 (clCasp3) on tumor sections of β-catenin^D164A/fl^ transgenic mice with Cre (D164A/-) or without Cre (D164A/fl) compared to control β-catenin^fl/fl^ mice without Cre expression (fl/fl). Each data point represents one histological section. Data are presented as mean ± SEM. Statistical analysis was performed using ordinary one-way ANOVA multiple comparison test. ns not significant.

We next assessed whether a mutation of the N-terminal domain in β-catenin, which abrogated the interaction with both Bcl9 and Bcl9L on the side of β-catenin, recapitulated the effects of B9/B9L-ΔHD2 in breast cancer progression. To this end, we employed knock-in mouse lines carrying a N-terminal point mutation abrogating the binding of Bcl9 and Bcl9l to β-catenin (D164A) [33]. Mice carrying one mutated allele and a conditional allele of β-catenin (designated as β-catenin^D164A/fl^ = D164A/fl) [33] were intercrossed with MMTV-PyMT mice and with MMTV-Cre mice. The resulting composite transgenic mice expressed β-catenin-D164A exclusively in mammary tumor cells (β-catenin^D164A/fl^;MMTV-PyMT;MMTV-Cre mice = D164A/−) (Fig. 6A). At 12 weeks of age, the composite transgenic mice expressing only the mutant forms of β-catenin showed a significantly lower tumor mass (Fig. 6B). The impairment of β-catenin binding to Bcl9/Bcl9L also significantly reduced tumor progression and lung metastasis formation (Fig. 6C–E). Notably, composite transgenic mice harboring one allele of the mutant forms and still one allele of a conditional (fl) but fully functional allele of β-catenin also showed reduced tumor growth and metastasis formation, indicating a dominant-negative effect of β-catenin-D164A as observed with the mutant forms of Bcl9/Bcl9L. Also here, we exclude a haploinsufficent effect of the loss of one allele of β-catenin, since tumor progression was not affected upon loss of only one allele of β-catenin in β-catenin^wt/fl^;MMTV-PyMT;MMTV-Cre mice as compared to β-catenin^wt/wt^;MMTV-PyMT;MMTV-Cre mice (Supplementary Fig. 5A).

Immunofluorescence microscopy analysis of tumor sections for pH3 as a marker for tumor cell mitosis and for clCasp3 as a marker of tumor cell apoptosis revealed that the reduced tumor growth in β-catenin mutant-expressing mice is due to diminished tumor cell proliferation (Fig. 6F and Supplementary Fig. 5B), and not to an increase in tumor cell apoptosis (Fig. 6G and Supplementary Fig. 5C). However, the expression of canonical Wnt signaling target genes c-Myc and Cyclin D1 known to regulate cell proliferation, like Axin 2 was not significantly affected by the abrogation of Bcl9/Bcl9L-β-catenin interactions in D164A cells (Supplementary Fig. 5D). Critically, the N-terminal mutation did not affect the interaction of β-catenin with E-cadherin: immunoprecipitation experiments (Supplementary Fig. 5E) and immunofluorescence microscopy analysis of co-localization at cell membranes (Supplementary Fig. 5F) demonstrated that the interaction with E-cadherin was retained by the D164 mutant form of β-catenin.

These data show that the interactions of Bcl9 and Bcl9L with β-catenin play a critical role in tumor cell proliferation, primary tumor outgrowth, malignant tumor progression, and metastasis formation.

### Bcl9/Bcl9L-Pygopus–β-catenin interactions modulate gene expression

**Fig. 7:**
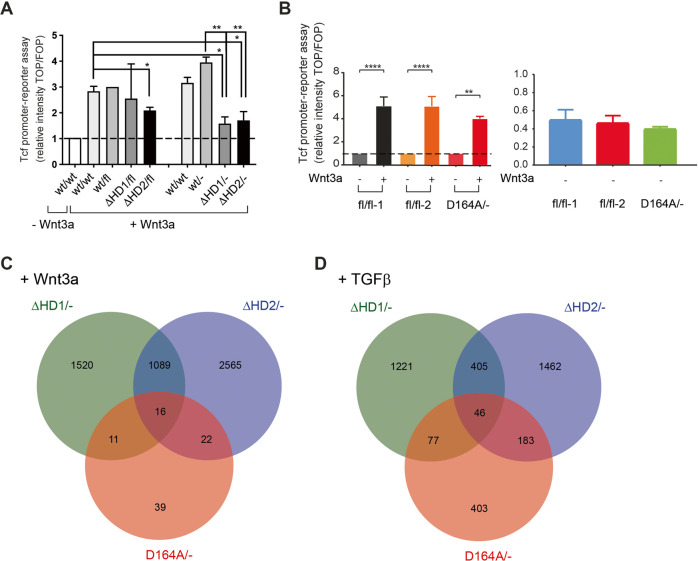
Comparative gene expression profiling of tumor-derived cell lines with defined B9/B9L and β-catenin mutations. **A**, **B** Primary epithelial tumor cells with B9/B9L wt/wt, wt/fl, ΔHD1/fl, ΔHD2/fl, and β-catenin fl/fl and D164A/fl genotypes were isolated from mammary tumors of the various genotype mice. B9/B9L wt/-, ΔHD1/- and ΔHD2/- and β-catenin D164A/- cell lines were generated by infection with Cre-expressing Adenoviruses (Ad-Cre-IRES-GFP). Cells with and without Cre expressions were treated with no cytokine or Wnt3a (3 days, 100 ng/ml). Tumor-derived cell lines expressing exclusively wild-type B9/B9L (wt/wt, wt/fl or wt/-) or exclusively B9/B9L mutants (ΔHD1/- and ΔHD2/-) (A) or exclusively wild-type β-catenin (fl/fl-1 and fl/fl-2) or β-catenin-D164A (D164A/-) (B) were transfected with superTOPflash (TCL/LEF-Firefly luciferase) or superFOPflash (mutated TCF-LEF-binding site) and treatment with or without Wnt3a for 3 days and canonical Wnt signaling output was determined by bioluminescence assays. Relative transcriptional output was calculated relative to wild-type cells in the absence of Wnt3a set to 1. The right panel in B shows the lack of variation of β-catenin/Tcf-mediated transcription in the various genotype β-catenin cell lines in the absence of Wnt3a. Statistical analysis was performed using ordinary one-way ANOVA multiple comparison test. *p < 0.05; **p < 0.01; ****p < 0.0005. **C**, **D** Cell lines derived from tumors of the various genotype MMT-PyMT transgenic mice were treated with recombinant Wnt3a (3 days; 100 ng/ml) or recombinant TGFβ (4 days; 2 ng/ml) and subjected to next-generation RNA-sequencing. Venn diagrams showing the overlap of differentially expressed genes between B9/B9L ΔHD1/-, B9/B9l ΔHD2/- and β-catenin D164A/- mutant cell lines compared to their wild-type control cell lines upon treatment with Wnt3a (C) and TGFβ (D). RNA changes with *p* ≤ 0.05 and a fold change ≥1.5 were considered differentially expressed. For the gene lists shared by the B9/B9L-β-catenin interaction mutants ΔHD2/- and D164/- please see Supplementary Tables I and II.

To examine the effects of ΔHD1, ΔHD2, and D164A knock-in mutations on Wnt/β-catenin and TGFβ-mediated transcriptional output we established cell lines derived from tumors with the defined B9/B9L and β-catenin genotypes. Floxed alleles of *Bcl9, Bcl9L*, and *β-catenin* genes were recombined by infection with Adenovirus expressing Cre-recombinase and GFP (Ad-Cre-IRES-GFP). Adenovirus-transduced cells were sorted by flow cytometry for GFP expression, and their genotype was confirmed by PCR analysis. Wnt3a-induced β-catenin-mediated transcriptional activity was then determined using a Super TOPflash/FOPflash promoter-reporter assay (TCF-LEF reporter). β-catenin/TCF-dependent transcriptional output was diminished in both ΔHD1/- and ΔHD2/- mutant cells (Fig. 7A), while β-catenin-D164A cells showed only a slight reduction in Wnt3a-induced transcriptional output as compared to two independent β-catenin-fl/fl cell lines expressing exclusively wild-type β-catenin (Fig. 7B).

These cell lines were then used for whole transcriptome analysis by RNA-sequencing and comparative gene expression profiling under three conditions: no cytokine control or treatments with either Wnt3a (100 ng/ml) for 3 days or TGFβ (2 ng/ml) for 4 days. The reason for the rather long-term cytokine treatment was based on the observation that profound cell morphology changes were best detected after such time spans (Supplementary Fig. 6A). ΔHD1/-, ΔHD2/-, and D164A/- mutant cell lines expressed exclusively B9/B9L-ΔHD1, B9/B9L-ΔHD2, or β-catenin-D164A, while ΔHD1/fl, ΔHD2/fl, and D164/fl mutant cell lines expressed B9/B9L-ΔHD1, B9/B9L-ΔHD2, or β-catenin-D164A and the floxed (wild-type) alleles. Hemizygous control wt/- cells expressed only one wild-type allele of *B9/B9L* genes, while fl/fl cells expressed two floxed alleles which behaved as wild-type alleles.

Unsupervised hierarchical clustering of the top 500 most variable genes between the various cell lines and the particular treatments revealed that the cell lines did not cluster according to their genotypes but rather to the treatments with Wnt3a or TGFβ (Supplementary Fig. 6B). This result suggests that the deficiencies in Bcl9/Bcl9L-Pygopus and β-catenin interactions were not dramatically affecting overall gene expression.

We thus analyzed which genes were differentially expressed upon treatment with Wnt3a or TGFβ and whether their expression was affected by the disruption of B9/B9L-β-catenin binding in the B9/B9L-ΔHD2 mutant and the β-catenin-D164A mutant cell lines. Only 38 Wnt3a-regulated genes were affected by the disruption of B9/B9l-β-catenin binding, while 229 TGFβ-regulated genes changed their expression upon loss of B9/B9l-β-catenin binding (Fig. 7C, D). Notably, these genes lists were not apparently enriched in any specific Wnt or TGFβ signaling target genes or any specific biological pathways (Supplementary Tables I and II). These results suggest that the interaction between Bcl9 and Bcl9L and β-catenin may be critical for the expression of a number of genes, yet that these genes may not specify particular biological processes. Hence, the subtle changes in gene expression may underlie the modulatory role of Bcl9 and Bcl9L on β-catenin-mediated Wnt signaling and tumor progression (see “Discussion”).

## Discussion

Most breast cancer deaths are due to the growth of metastatic cells in vital organs. Both canonical Wnt and TGFβ-signaling are key pathways in the regulation of cellular state, cancer cell proliferation, and cancer cell invasion. In breast cancer the components of both pathways are rarely mutated, yet both pathways are frequently found activated in malignant and metastatic breast cancer [8, 34]. The functional interactions between these morphogenic signaling pathways are only poorly understood during the process of an EMT and the malignant progression and metastasis of breast cancer [4, 5, 29].

Here, we have dissected the roles of the interactions of Bcl9 and Bcl9L with Pygopus and with β-catenin during tumor progression and metastasis formation in the MMTV-PyMT mouse model of metastatic breast cancer. We have employed mouse lines carrying conditional alleles of B9/B9L and knock-in mouse lines carrying various alleles of B9/B9L and of β-catenin: B9/B9L-ΔHD1 lack the capability to bind to Pygopus [13], B9/B9L-ΔHD2 fail to bind to β-catenin [13], and β-catenin-D164A cannot bind to B9/B9L [33].

We found that the loss of Pygopus 2-B9/B9L binding in cancer cells expressing the ΔHD1 mutant forms of Bcl9/9L results in smaller tumors and less metastases. This result is consistent with our recent report demonstrating that the loss of Pygous 2 interaction with histones in tumors of MMTV-PyMT transgenic mice resulted in smaller and more differentiated tumors with less metastasis [35]. However, we did not see a significant reduction of tumor cell proliferation or increase in apoptosis, as expected from the reported role of Pygopus 2 in cell proliferation [36].

Interfering with the β-catenin-B9/B9L interactions with Bcl9/9L-ΔHD2 or β-catenin-D164A provoked a significant reduction in primary tumor growth, malignant tumor progression, and metastasis formation. This was mainly due to diminished tumor cell proliferation and increased tumor cell apoptosis. The more profound effects observed upon loss of B9/B9L binding to β-catenin, compared to a loss of binding to Pygopus, are consistent with a recent report demonstrating that B9/B9L sustains β-catenin transcriptional activity in colorectal cancer cells and in developing forelimbs in a Pygopus-independent manner [18]. Notably, all mutant versions of B9/B9L and β-catenin exerted a dominant-negative effect on tumor growth and metastasis formation, suggesting that the interaction mutants disrupted functional β-catenin transcriptional complexes.

Altogether, the results indicate that the binding of Bcl9/9L to β-catenin and Pygopus is important for malignant breast cancer progression and metastasis formation. This is consistent with several reports that B9/B9L-deficiency diminishes tumor load and to decrease EMT and metastasis formation in other mouse cancer models: myeloma, intestinal cancer, colorectal cancer, non-small cell lung cancer, hepatocellular carcinoma, and pancreatic adenocarcinoma [19, 20, 37–41]. In this context, it is important to note that canonical Wnt signaling is known to promote cell proliferation [42, 43], and in our experiments the specific ablation of the binding of B9/B9L to β-catenin distinctly affects tumor growth and tumor progression. This suggests that the Bcl9/Bcl9L-β-catenin interaction is critical for tumor progression.

These results stand in contrast to the observation that the complete loss of B9/B9L expression induces apoptosis of cultured breast cancer cells in vitro. Moreover, in the MMTV-PyMT mouse model of metastatic breast cancer, B9/B9l-deficient cells were selected against, and primary tumor growth and metastasis were reconstituted by cancer cells which had not been genetically depleted for B9/B9L (aka escape phenotype). Apparently, the complete loss of Bcl9/Bcl9L impairs survival of mammary tumor cells, while the disruption of the β-catenin-Bcl9/Bcl9L-Pygopus chain of adapters only affects the tumorigenic behavior of the tumor cells. These results may suggest that Bcl9 and Bcl9L may exert β-catenin-independent functions, a notion which warrants further investigations, as previously described [13].

Since the RNA-sequencing of cell lines expressing the mutant forms of B9/B9L and β-catenin did not reveal major changes, we postulate the interaction is critical for fine-tuning Wnt/β-catenin signaling during malignant breast cancer progression. In particular, we could not identify Wnt and TGFβ-specific gene signatures. It rather appeared that the Bcl9/Bcl9L-β-catenin binding deficiency caused a moderate decrease of the expression of genes of various pathways. Thus, Bcl9 and Bcl9L may promote mammary tumor growth and metastasis formation in MMTV-PyMT mice in a rather moderate manner and by fine-tuning the expression levels of direct and indirect Wnt target genes. These results are reminiscent of other studies where the disruption of Bcl9 and Bcl9L functions had no effect on normal tissue homeostasis, but moderately repressed carcinogenesis, an observation consistent with the “just-right” hypothesis of Wnt-driven tumor formation [39, 40]. Hence, the question remains whether Bcl9 and Bcl9L aid in the selection of specific β-catenin target genes or modulate the quantitative difference in β-catenin-dependent transcriptional output on specific target genes. Along these lines, loss of the specific B9/B9L/β-catenin/Pygopus interactions also affected the TGFβ-mediated signaling output. A functional interaction between Wnt and TGFβ signaling during EMT and other pathophysiological processes has been demonstrated before [42–44]. Our results underscore the importance of this crosstalk. However, a more complete picture is missing because of the challenges of identifying specific Wnt and TGFβ target genes in different cell types and in varying cell contexts.

Analysis of the expression of Bcl9 and Bcl9L revealed that both proteins are highly expressed during mammary tumor progression in MMTV-PyMT transgenic mic. Database mining indicated that *BCL9* and only to a lesser extent *BCL9L* are found mutated in breast cancers of patients. *BCL9* gene amplification appeared to be the most frequent genetic alteration in breast cancer with highest levels of amplifications in basal type and lowest in normal-type breast cancers. However, *BCL9L* gene alterations correlated with poor disease and progression-free survival of patients. Altogether, these results support the notion that BCL9 and BCL9L are critically players in breast carcinogenesis. These results are also consistent with previous work identifying nuclear BCL9 as a molecular driver in DCIS invasive progression [21]. Moreover, it has been reported that BCL9L is a critical player in regulating estrogen receptor expression in breast cancer, notably, in a β-catenin independent manner [31]. In fact, the mammary epithelial cell-specific transgenic expression of Bcl9L is sufficient to induce ductal-like mammary tumors in aged mice [31].

The increased expression of BCL9 and BCL9L may be caused by downregulation of microRNAs, such as mir30a [45, 46], miR-30c-2 [47], mir122 [48], mir128 and miR-1301 [49]. BCL9L expression has also been reported to be regulated by transcriptional repression by the tumor suppressor WWOX and HDAC3-mediated histone acetylation [50]. Another mechanism is suggested by the observation that the expression of *BCL9* can be induced by hypoxia in human hepatocellular carcinoma: BCL9 levels can also be stabilized by the deubiquitinase USP9X in a variety of cancer cell lines. Increased BCL9 levels in these cell lines promote the formation of the β-catenin transcriptional complex and the expression of Wnt target genes [45, 51]. However, it should also be noted that β-catenin-independent functions of BCL9 have been reported, such as in regulating mRNA stability of calcium signaling and neural-associated genes and thus promoting colorectal carcinogenesis [52].

Altogether, our results indicate that the interactions of B9/B9L with β-catenin, and to a lesser extent with Pygopus, represent a critical mechanistic contribution to Wnt signaling-mediated malignant tumor progression and metastasis formation and, thus, may offer attractive opportunities to therapeutically interfere with canonical Wnt signaling and with malignant tumor progression. Indeed, first biological and pharmacological approaches have been reported which prevent or disrupt the interactions between B9/B9L and β-catenin. For example, the Hippo signaling pathway kinase LATS2 has been reported to disrupt BCL9-β-catenin interaction in a kinase-independent function in colorectal cancer cells [53]. Subsequently, specific peptides have been designed to disrupt B9/B9L-β-catenin interactions and proven to block Wnt-driven cancer cell proliferation and to induce apoptosis [54, 55]. On the other hand, the interaction of β-catenin with B9/B9L appears to protect β-catenin from Tankyrase inhibitor-induced degradation [56]. In conclusion, targeting B9/B9L-β-catenin interactions may carry great potential in interfering with any cancer type that is driven by canonical Wnt signaling, including breast cancer. However, whether the specific targeting of B9/B9L-β-catenin interactions will impair Wnt-driven organ homeostasis and thus cause major side effects of cancer therapy, needs to be investigated.

## Material and methods

See also Supplementary Material and Methods.

### Mouse experiments

Mouse colonies were kept at the animal facility of the Department of Biomedicine, University of Basel, Switzerland. All animal experiments were carried out in accordance with the guidelines of the Swiss Federal Veterinary Office and the Cantonal Veterinary Office of Basel-Stadt. To examine the conditional ablation of Bcl9/Bcl9L in breast cancer cells, Bcl9^fl/fl^ and Bcl9L^fl/fl^ mice [19] were crossed with MMTV-PyMT (a kind gift of N. Hynes, FMI, Basel, Switzerland) [24, 57] and MMTV-Cre (a kind gift of Lothar Hennighausen, NHI, Bethesda, USA) mice [32]. Bcl9/Bcl9L^fl/fl^;MMTV-PyMT, and MMTV-Cre mice were then crossed with Bcl9/Bcl9L^ΔHD1/wt^ or Bcl9/Bcl9L^ΔHD2/wt^ [13] or β-catenin^D164A/wt^ [33] knockin mouse lines to generate mice. All mouse lines were maintained in an FVB/N genetic background, and experimental mice were always compared to littermate controls. To monitor MMTV-Cre-mediated recombination, Bcl9/Bcl9L^fl/fl^;MMTV-PyMT;MMTV-Cre mice were crossed with GFP-reporter mice (R26-LSL-GFP, kindly provided by V. Taylor, DBM, Basel, Switzerland). For the quantification of recombination, whole tumor sections were imaged with a Zeiss Axio Imager Scanning Microscope (×10 magnification). GFP-positive areas and total tumor areas (DAPI-positive area) were quantified using ImageJ. All experiments were performed with female mice. For analysis of the mammary gland, mice were sacrificed at 5 weeks and before a tumor volume of 1500 mm^3^ was reached, usually at 12–13 weeks of age.

### Cell and tumor genotyping

To extract genomic DNA, cells from a confluent 10 cm petri dish were trypsinized, washed in PBS, and pelleted by centrifugation. DNA was extracted using GenElute™ Mammalian Genomic DNA Miniprep Kits (G1N70, Sigma-Aldrich) according to the manufacturer’s protocol. Standard PCR was performed using the following primers: for the β-catenin floxed and mutant allele, sense primer RM41 (5′-AAG GTA GAG TGA TGA AAG TTG TT-3′) and antisense primer RM42 (5’-CAC CAT GTC CTC TGT CTA TTC-3′) were used, generating 324 and 221 bp products from the floxed and mutant alleles, respectively. To detect the floxed allele, sense primer RM68 (5′-AAT CAC AGG GAC TTC CAT ACC AG-3′) and antisense primer RM69 (5′-GCC CAG CCT TAG CCC AAC T-3′) were used generating a 631 bp product from the deleted allele [58].

Genotyping Bcl9 loxP site (expected band: 350 bp for loxP and 265 bp WT)

Bcl9 forward primer: (5′-CCTGCCGAGATGGTCTCAGTTC-3′)

Bcl9 reversed primer: (5′-CACCCCAGGCTACCTCACTGAC-3′)

Genotyping Bcl9L loxP site (expected band: 640 bp for loxP and 560 bp WT)

Bcl9L forward primer: (5′-CAACCCACCGGGACCTCTC-3′)

Bcl9L reversed primer: (5′-GGAGGAGCGGAGGAGCTGTTC-3′)

Genotyping Bcl9 WT and recombined (expected band: 284 bp for recombined; 162 bp for WT, and 400 bp for Lox)

Bcl9 D1 forward primer: 5′-CCACCAAGGAATCGCAGACGTG-3′

Bcl9 D2 reversed primer 1: 5′-TGCAACTGAGCTGGGATGTTTGC-3′

Bcl9 D3 reversed primer 2: 5′-GCTGGGCCCATGCTTGCTC-3′

Genotyping Bcl9l WT and recombined (expected band: 345 bp for recombined; 260 bp for WT)

Bcl9L D1 forward primer: 5′-TGTCCTCCCTACCTTCCCCTTGG-3′

Bcl9L D2 reversed primer 1: 5′-GCGAGGTTAACGTCCCCCAAATC-3′

Bcl9L D3 reversed primer 2: 5′-TTGCTCAAGATGGCCAGGATGC-3′

### Cell line derivation

Cells were isolated from thoracic mammary glands with tumors lesions of 12-week-old MMTV-PyMT transgenic female mice (FVB/N background). Small tumor pieces were minced, and 10 ml of predigestion buffer (10 mM Hepes pH 7.4, 14 mM NaCl, 0.67 mM KCl, 1 mM EDTA supplemented with 50 g/ml gentamycin (Sigma, G1397) and 1X antibiotic-antimycotic (15240062, Thermo Fisher Scientific) were added and incubated for 30 min at 37 °C on a shaker. After washing with PBS, the samples were digested using 6 ml digestion buffer (10 mM Hepes pH 7.4, 142 mM NaCl, 0.67 mM KCl, 0.67 mM CaCl_2_, 20 mM Glucose supplemented with 1 mg/ml Collagenase D (Roche, 11088858001), 50 g/ml gentamicin (Sigma-Aldrich, G1397) and 1X antibiotic-antimycotic (15240062, Thermo Fisher Scientific) and incubated for 30 min at 37 °C on a shaker. Subsequently the samples were washed with PBS and then resuspended in growth medium and seeded in 10 cm plates. The medium was changed regularly and fibroblasts in culture were removed by differential trypsinization until only epithelial cells remained. The cell lines were further cultured in DMEM supplemented with glutamine, penicillin, streptomycin, and 10% heat-inactivated FBS (F7524, Sigma-Aldrich) and 10% horse serum (Amimed). To establish cell lines only expressing the mutant alleles, cells carrying one floxed allele and one mutant allele were seeded into 10 cm plates and on the next day infected either with Adeno-CRE-IRES-GFP or with the Adeno-IRES-GFP virus as control using FuGENE^®^ HD Transfection Reagent (E2311, Promega). Next day, the medium was changed, and after 3 days the cells were sorted for GFP-positive cells into 24-well plates using a BD FACSAria Flow Cytometer (BD Biosciences). Detachment of the cells was performed using trypsinization followed by two-times washing in 1x PBS and resuspension in 2% FBS, PBS and syringe filtering (40 µm mesh filter) immediately before FACS sorting into a polystyrene round bottom tube (352054, FALCON) filled with DMEM medium. After centrifugation, supernatants were discarded, and cells were resuspended and seeded into 24-well plates.

### Adenovirus infection

Cells were plated onto 6 cm dishes in duplicates and transfected the next day with Adeno-CRE-IRES-GFP or with Adeno-IRES-GFP virus as a control (1710 and 1761, Vector Biolabs) using Fugene HD transfection reagent (E2311, Promega). The following day, medium was changed and cell culture continued.

### Luciferase reporter assay

The firefly luciferase reporter constructs superTOPflash and superFOPflash (kindly provided by Konrad Basler, UZH Zürich) were used to quantify Wnt/β-catenin-mediated transcriptional output. Cells were plated in duplicates in 24-well plates and transfected the following day with 500 ng of the superTOPflash or superFOPflash *firefly* luciferase reporter plasmid and 10 ng of a constitutive-active *Renilla* luciferase plasmid using Lipofectamine 3000 Reagent (Invitrogen) according to the manufacturer’s instructions. Luciferase activities were measured using the Dual-Luciferase® Reporter Assay System (E1960, Promega). *Firefly* luciferase values were normalized to *Renilla* luciferase control values.

For Smad and TCF reporter assays, we used the Smad reporter, TCF reporter and the Negative Control from the Cignal 45-Pathway Reporter Array (Qiagen, Lenti Reporter Assays). This assay is based on dual-luciferase technology and Negative control serves as a specificity control. The Smad reporter consists of Smad2/3/4 transcription factors-responsive firefly luciferase construct and a constitutively expressing Renilla luciferase construct. Cells were transduced with lentiviral particles for assessing canonical TGFβ activity, which is determined by comparing the normalized luciferase activities of the reporter in treated versus untreated cells.

### siRNA transfections

NMuMG/E9, Py2T, and Py2T-LT cells (TGFβ **>** 20 days) were transfected with Silencer select siRNAs from Ambion (Thermo Fisher Scientific) using Lipofectamine RNAiMAX (Thermo Fisher Scientific). Three oligoes against Bcl9 and Bcl9L were tested: siB9 (Oligo A, B, and C: s95054, s95055, and s95056) and siB9L (Oligo A, B, and C: s95949, s95950, and s95951). In addition, we tested siRNA pools against Bcl9 and Bcl9L and Smad4 from Dharmacon (SmartPool ON-Target Plus). For the study of EMT induction, cells were transduced with specific siRNAs 2 days before starting with TGFβ treatment (Py2T-LT is continuously under TGFβ). For EMT induction with 4 days of TGFβ treatment, cells were re-transfected at day 3 for NMuMG/E9 (20 nM each) and at days 3 and 5 for Py2T cells (40 nM each) to ensure proper siRNA-mediated gene downregulation until the end of the assay on day 6. Cells are then used for immunofluorescent staining, migration assay or for the isolation of RNA (Trizol) for quantitative RT-PCR.

### Migration assays

Py2T-LT cells (TGFβ **>** 20 days) were transfected for 2 days with siRNA against Bcl9 and/or Bcl9L or control siRNA (siCtr). In total, 2.5 10^4^ cells were plated onto a 0.2–20% FBS/DMEM Gradient medium in a 24-well Boyden chamber plate with 8.0 μm Transparent PET Membrane (Corning, NY, USA) and incubated for 18 h at 37 °C. Cells on the membrane were fixed with 4% paraformaldehyde and counterstained with DAPI (1 μg/ml; Sigma D9542). Non-migrated cells from the upper surface of the transwell membrane were removed by scrapping with a cotton swab. Migrated cells were imaged from the bottom of the Transwell membrane with a 10x objective on a Leica DMI microscope and quantified with ImageJ software.

### RNA-sequencing and analysis

Established cell lines (B9/B9L^wt/wt^, B9/B9L^wt/fl^, Bcl9/Bcl9L^ΔHD2/fl^, β-catenin^fl/fl^, and β-catenin^D164A/fl^) with and without *floxed alleles* were treated either with 100 ng/ml Wnt3a for 3 days or with 2 ng/ml TGFβ for 4 days. Untreated cells served as control. Biological duplicates were prepared, total RNA was isolated using the miRNeasy Mini Kit (Qiagen, 217004) with on-column DNAse digestion according to the manufacturer’s instructions. RNA quality control was performed using RNA ScreenTape on an Agilent 4200 TapeStation, and RNA concentration was measured using Quanti-iT RiboGreen RNA assay Kit (Life Technologies). RNA-sequencing libraries were prepared from total RNA using poly(A) enrichment using 200 ng input RNA with TruSeq stranded mRNA Sample prep (Illumina). QC was performed on a fragment analyzer using DNF-473-33-SS NGS Fragment 1–6000 bp kit. RNA sequence libraries were sequenced on a NextSeq 500 using 75 cycles kit High Output (Illumina). Single-end RNA-sequencing reads were mapped to the mouse genome assembly, version mm10, using RNA-STAR (PMID:23104886), with default parameters except for allowing only unique hits to genome (outFilterMultimapNmax **=** 1) and filtering reads without evidence in spliced junction table (outFilterType **=** “BySJout”). Using RefSeq mRNA coordinates from UCSC (genome.ucsc.edu, downloaded in December 2015) and the qCount function from QuasR package (version 3.12.1) (PMID:25417205) we quantified gene expression as the number of reads that started within any annotated exon of a gene. The differentially expressed genes were identified using the edgeR package (version 1.10.1) (PMID:19910308). Genes with fdr ≤0.05 and minimum log2 fold change of ±1.0 were considered statistically significant and included in further analysis.

### RNA integrity and quantification

RNAs were quality-checked on the TapeStation instrument (Agilent Technologies, Santa Clara, CA, USA) using the RNA ScreenTape (Agilent, Cat# 5067-5576)—Average RINewas 9.7. RNA were quantified by Fluorometry using the QuantiFluor RNA System (Cat# E3310, Promega, Madison, WI, USA).

### Library preparation

Library preparation was performed, starting from 200 ng total RNA, using the TruSeq Stranded mRNA Library Kit (Cat# 20020595, Illumina, San Diego, CA, USA) and the TruSeq RNA UD Indexes (Cat# 20022371, Illumina, San Diego, CA, USA).

Libraries were quality-checked on the Fragment Analyzer (Advanced Analytical, Ames, IA, USA) using the Standard Sensitivity NGS Fragment Analysis Kit (Cat# DNF-473, Advanced Analytical) revealing excellent quality of libraries (average concentration was 129 ± 14 nmol/l and average library size was 350 ± 11 base pairs).

Samples were pooled to equal molarity. The pool was quantified by Fluorometry using the QuantiFluor ONE dsDNA System (Cat# E4871, Promega, Madison, WI, USA).

### Clustering and sequencing

Libraries were sequenced Single-reads 101 bases (in addition: 8 bases for index 1 and 8 bases for index 2) using the NovaSeq 6000 instrument (Illumina) and the S1 Flow-Cell loaded at 400pM and including 1% PhiX.

Primary data analysis was performed with the Illumina RTA version V3.4.4. Two Novaseq S1 Flow-Cells runs were performed to compile enough reads (on average per sample: 34.8 ± 3.2 millions pass-filter reads).

### Human data analysis

cBioportal online tool for cancer Genomics (https://www.cbioportal.org/; 10.1158/2159-8290.CD-12-0095, 10.1126/scisignal.2004088) is used for visualization and analyzing of the genetic alteration of BCL9 and BCL9L genes in breast cancer patient data (from TCGA-BRCA PanCancer Atlas data collection). In addition, we analyzed the enrichment of BCL9 and BCL9L gene amplifications in different cancer subtypes and correlation of these gene amplifications on the decease progression in breast cancer patients.

### Statistical analysis

Statistical analysis and graphs were generated using GraphPad Prism 7.02 software. All data are presented as mean ± SEM.

## Supplementary information

Supplementary Information

Supplementary Table I

Supplementary Table II

## Data Availability

The RNA-sequencing data are deposited on GEO database under GSE148843 and GSE182404.
