# Supplementary material for: The interactions of Bcl9/Bcl9L with β-catenin and Pygopus promote breast cancer growth, invasion, and metastasis
Source: Oncogene. 2021 Sep 20;40(43):6195–209. doi: 10.1038/s41388-021-02016-9 (PMC8553620; doi:10.1038/s41388-021-02016-9)
Supplement: Supplementary file 1 — Supplementary Information [file 41388_2021_2016_MOESM1_ESM.pdf]

Supplementary Information

The specific interactions of Bcl9/Bcl9L with β-catenin and Pygopus promote breast cancer growth, invasion and metastasis

Vida Vafaizadeh, David Buechel, Natalia Rubinstein, Ravi K.R. Kalathur, Meera Saxena, Tomas Valenta, George Hausmann, Claudio Cantù, Konrad Basler, and Gerhard Christofori

Supplementary Figures

Suppl. Figure 1; Vafaizadeh et al.

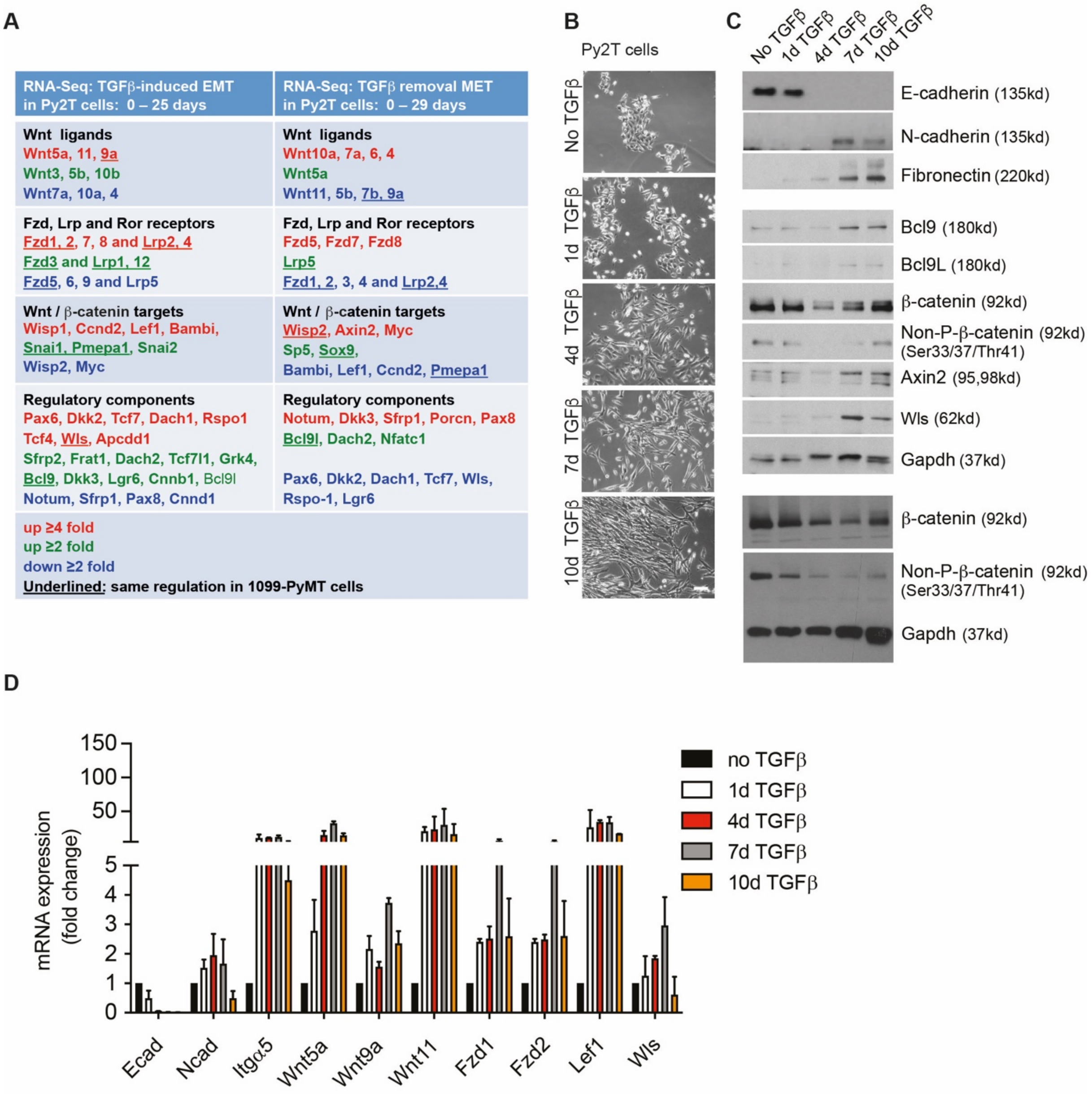

Suppl. Figure 1. Wnt-β-catenin signaling is activated during TGFβ-induced EMT.

(A) Differential gene expression analysis of Wnt signaling pathway components and Wnt signaling targets during a time course of TGFβ-induced EMT and its reverse process, MET, upon withdrawal of TGFβ in murine breast cancer cells. RNA-sequencing data of distinct time points of time courses of EMT and MET of the epithelial mammary tumor cell lines Py2T and PyMT-1099 [27, 29] was analyzed for changes in gene expression.

(B) Phase contrast microscopy imaging of the morphological changes of Py2T cells during a time course of TGFβ treatment of Py2T cells. Scale bar, 100μm.

(C) Immunoblotting analysis of Py2T cells induced to undergo EMT by TGFβ and analyzed after 0, 1, 4, 7 and 10 days of TGFβ treatment (2ng/ml). Dynamic changes in protein levels and changes in phosphorylation levels of some components of canonical Wnt signaling are shown during TGFβ-induced EMT in Py2T cells. The bottom part of the panel shows a repeat of immunoblotting for non-phosphorylated (active) and total β-catenin to demonstrate that activated β-catenin already exists in epithelial cells, is reduced during the early stages of EMT and re-appears at the later stages of EMT.

(D) Changes in the expression of WNT ligands, Wnt signaling components and epithelial and mesenchymal markers during TGFβ-induced EMT. Py2T cells were treated with TGFβ (2ng/ml) for 0, 1, 4, 7 and 10 days, and gene expression was analyzed by quantitative RT-PCR.

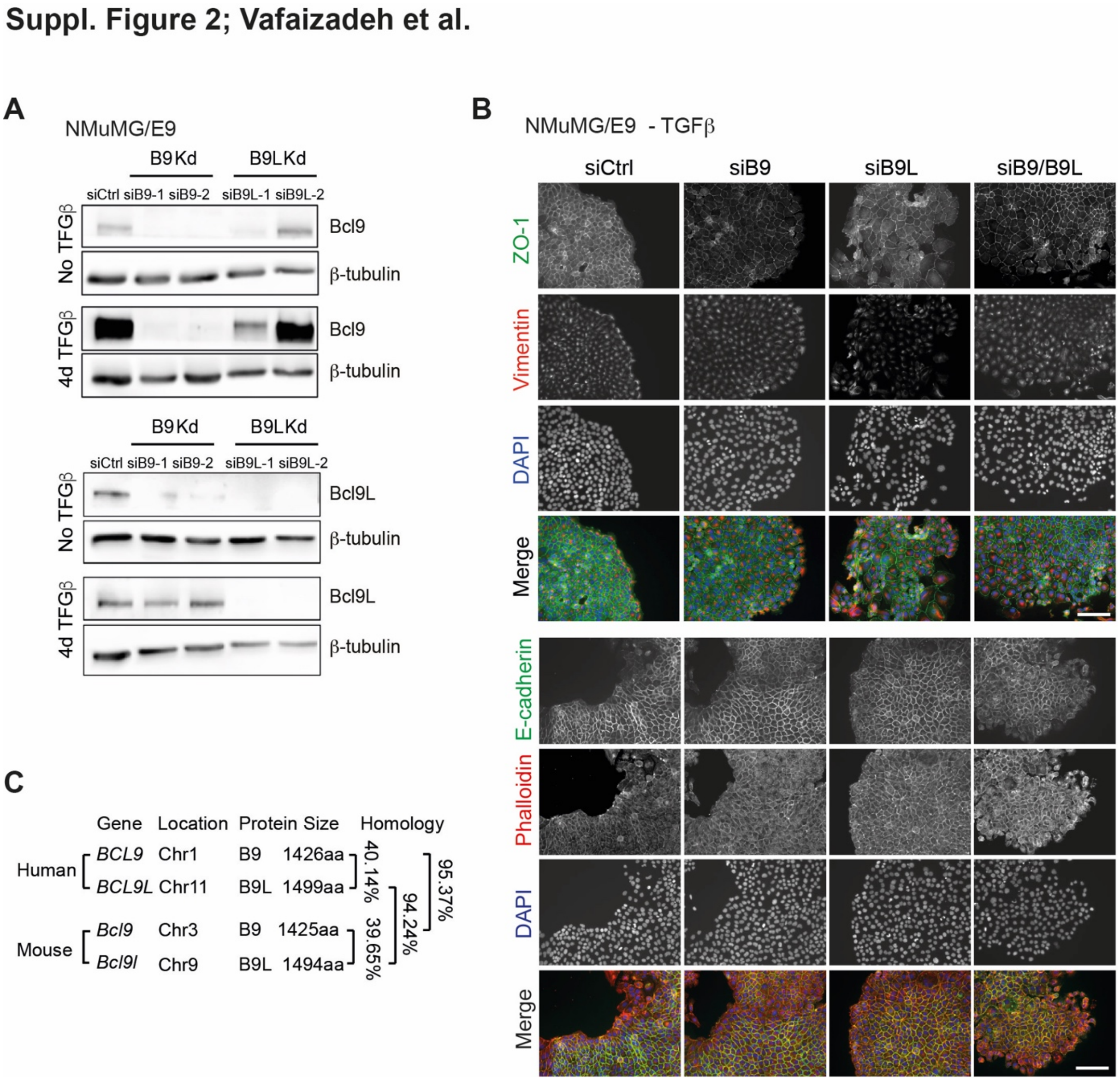

Suppl. Figure 2. Bcl9/Bcl9L contribute to TGFβ-induced EMT.

(A) Immunoblotting analysis of Bcl9 and Bcl9L protein levels in NMuMG cells transfected with siCtrl, siB9 or siB9L in the absence or in the presence of TGFβ for 4 days. siB9-2 and siB9L-2 showed more specificity and were used for further experiments.

(B) Depletion of Bcl9 and Bcl9L prevents TGFβ-induced EMT. NMuMG/E9 cells were transfected with control siRNA (siCtrl) or siRNAs against *Bcl9* (siB9), *Bcl9L* (siB9L) or both (siB9/B9L), but were not stimulated with TGFβ. Immunofluorescence microscopy analysis visualized the epithelial markers tight junction protein-1 (ZO-1) and E-cadherin and the mesenchymal marker Vimentin. Fluorescently labeled phalloidin visualized the actin cytoskeleton, and nuclei were counterstained with DAPI. The expression of ZO-1, Vimentin and DAPI are also shown in siRNA-transfected NMuMG/E9 cells with no TGFβ treatment

(lower fluorescent images). Scale bar, 100µm.

(C) *BCL9* and *BCL9L* genes display a high degree of homology between human and mouse, and the proteins are highly conserved between both species.

Suppl. Figure 3; Vafaizadeh et al.

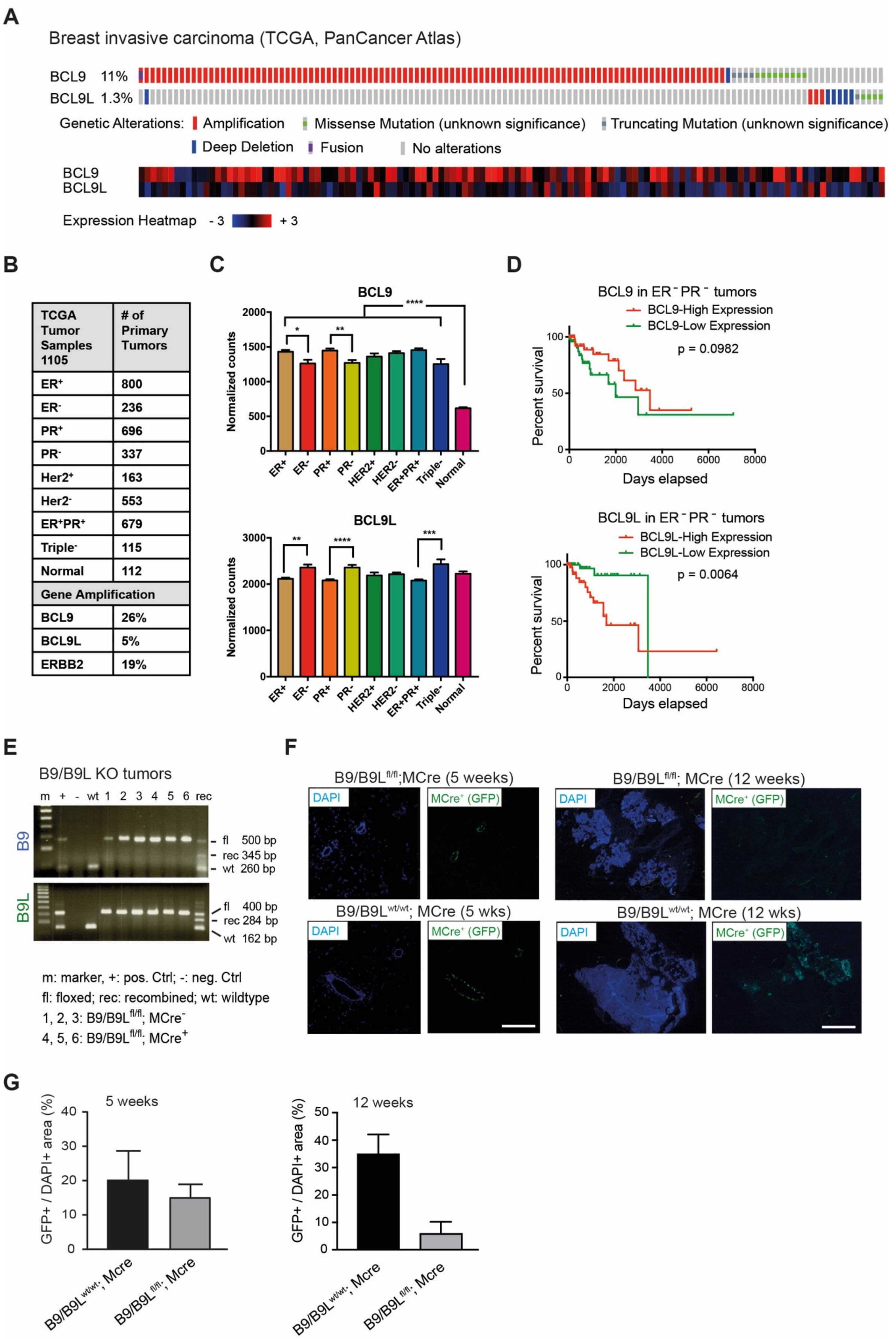

Suppl. Figure 3. Bcl9 and Bcl9L are highly expressed in invasive breast cancers.

(A) Analysis of genetic alterations in the *BCL9* and *BCL9L* genes across 1084 patients with breast cancer (from TCGA-BRCA PanCancer Atlas data collection) revealed a high frequency of *BCL9* gene amplification. The heatmap at the bottom of the panel shows *BCL9* and *BCL9L* gene expression in breast cancer patients with genetic alterations (cBioportal online tool).

(B) Number of primary tumor samples of the various subtypes of breast cancers available at TCGA database and used for the analysis of gene amplifications of *BCL9*, *BCL9L* and *ERBB2* (Her2).

(C) Normalized expression levels of BCL9 and BCL9L in the TCGA human breast cancer samples listed in (A).

(D) Kaplan Meyer correlation curves between the overall survival and the gene expression levels of BCL9 (upper panel) and BCL9L (lower panel) in patients with estrogen receptor (ER) and progesterone receptor (PR)-negative breast cancers in the TCGA database.

(E) Genotyping of primary tumors in MMTV-PyMT mice carrying wildtype (wt), floxed (fl), or recombined (rec) alleles of Bcl9 (B9) and Bcl9L (B9L) tumors and without or with the expression of Cre recombinase ($MCre^-$ or $MCre^+$) as determined by PCR and agarose gel electrophoresis.

(F) Efficiency of MMTV-Cre-mediated recombination. $B9/B9L^{wt/wt}$;Mpy;MCre and $B9/B9L^{fl/fl}$;Mpy;MCre mice were crossed with GFP-reporter mice (R26-LSL-GFP). GFP-positive areas ($MCre^+$) and total tumor areas ($DAPI^+$) were then visualized by immunofluorescence microcopy on whole tumor sections at 5 or 12 weeks of age of the mice. Scale bars, 100µm.

(G) Quantification of Cre-mediated recombination (GFP+ cells) in tumors of 5 and 12-week-old mice as described in (F).

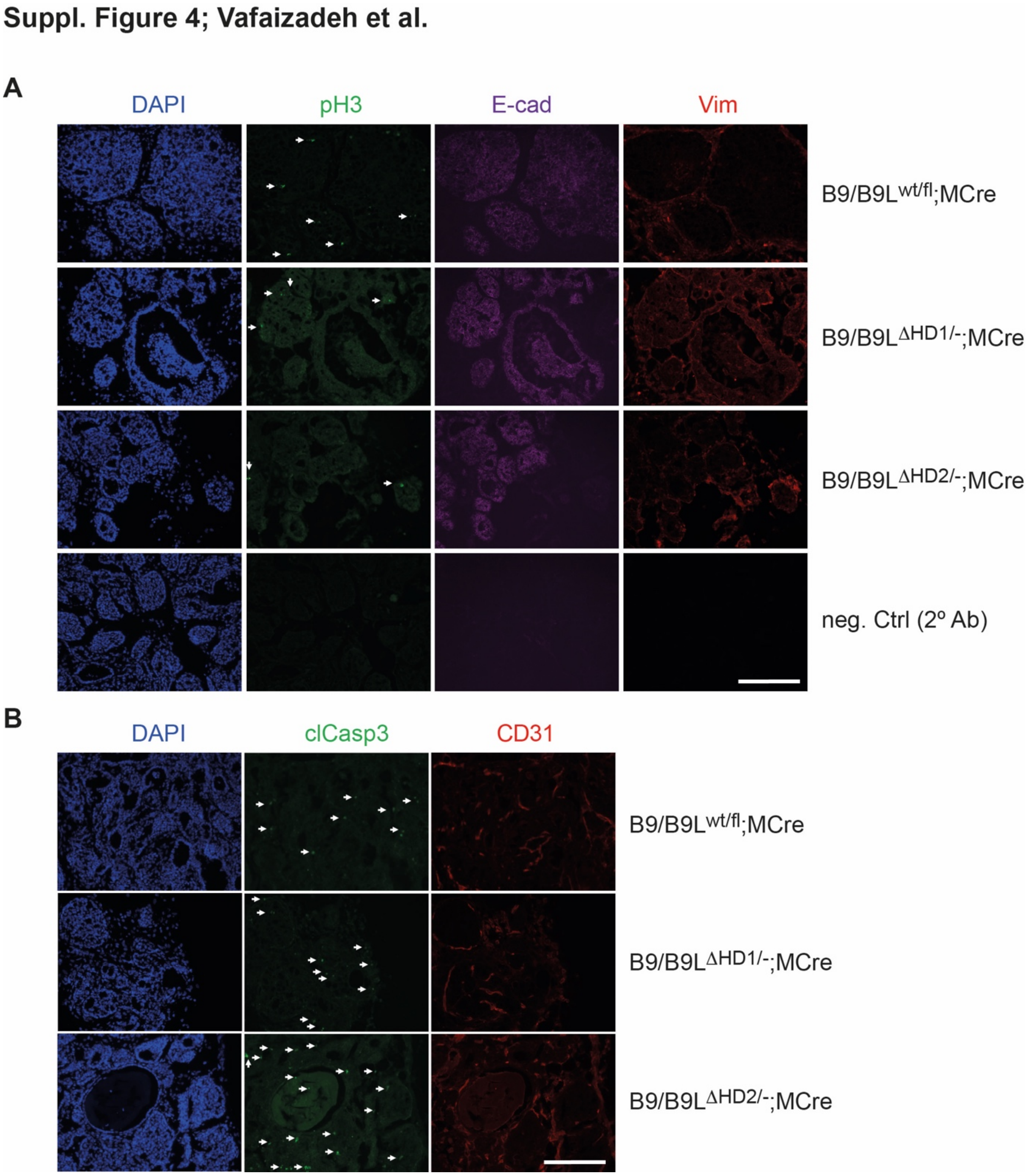

Suppl. Figure 4. Impact of disrupting the interaction of B9/B9L with Pygopus or with β-catenin on tumor cell mitosis and apoptosis.

(A) Tumor cell mitosis was determined by staining for phospho-histone 3 (pH3) on tumor sections of mice expressing wildtype BCl9 and Bcl9L (BB9/B9L wt/fl;MCre), the ΔHD1 mutant forms of Bcl9 and Bcl9L (BB9/B9L$^{\Delta HD1;}$MCre), or the ΔHD2 mutant forms of Bcl9 and Bcl9L (BB9/B9L$^{\Delta HD2;}$MCre), marked by arrows. Staining for Vimentin and E-cadherin was

performed to monitor the expression of epithelial vs. mesenchymal markers. DAPI staining visualizes cell nuclei. neg. Ctrl (2ºAb) = negative control with secondary antibody only. Scale bar, 100µm.

(B) Immunofluorescence staining of tumor sections for the early apoptosis marker cleaved Caspase-3 (clCasp3) on tumor sections of mice expressing wildtype (BB9/B9L wt/fl;MCre), the ΔHD1 mutant forms of Bcl9 and Bcl9L (BB9/B9L$^{\Delta HD1;}$MCre), or the ΔHD2 mutant forms of Bcl9 and Bcl9L (BB9/B9L$^{\Delta HD2;}$MCre), marked by arrows. Immunofluorescence staining for CD31 marks blood capillaries, and DAPI visualizes cell nuclei. Scale bar, 100µm.

Suppl. Figure 5; Vafaizadeh et al.

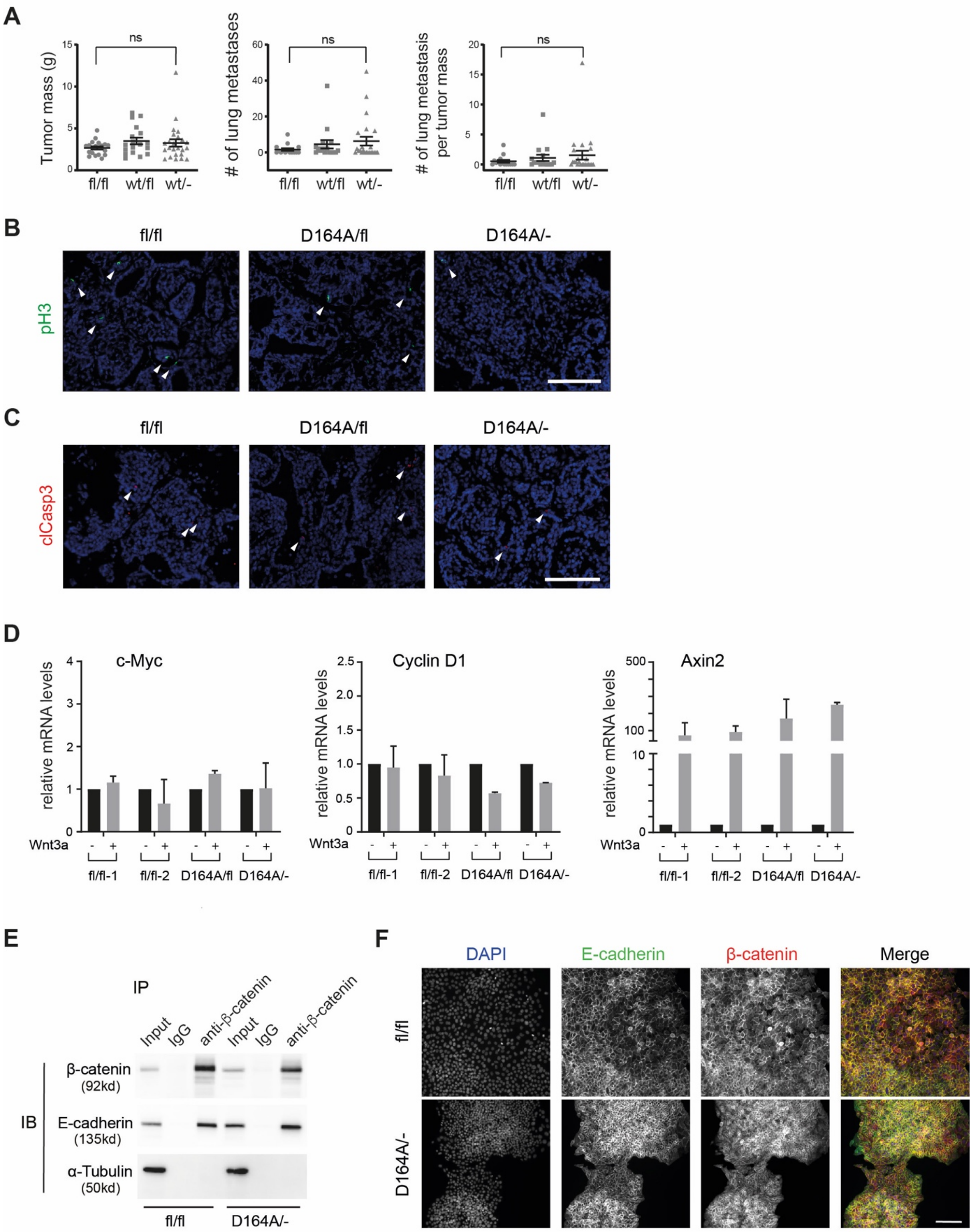

Suppl. Figure 5. Impact of disrupting the interaction of β-catenin with Bcl9/Bcl9L on tumor cell mitosis and apoptosis.

(**A**) Loss of one allele of β-catenin does not affect primary tumor growth and metastasis formation. Cre recombinase-mediated ablation of one allele of β-catenin in β-catenin$^{wt/fl}$;MMTV-PyMT;MMTV-Cre triple-transgenic mice did not affect primary tumor growth and lung metastasis formation as compared to mice expressing both wildtype alleles of β-catenin. Mouse numbers analyzed for primary tumor growth: β-catenin$^{fl/fl}$ (fl/fl), n=19; β-catenin$^{fl/wt}$ (wt/fl), n=18; β-catenin$^{fl/wt}$;MMMTV-Cre (wt/-). Mouse numbers analyzed for lung metastasis formation: β-catenin$^{fl/fl}$ (fl/fl), n=15; β-catenin$^{fl/wt}$ (wt/fl), n=16; β-catenin$^{fl/wt}$;MMMTV-Cre (wt/-), n=23. Data are displayed as mean ± SEM. Statistical analysis was performed using ordinary one-way ANOVA multiple comparison test. ns, not significant.

(B) Tumor cell mitosis was determined by immunofluorescence staining for phospho-histone 3 (pH3) on tumor sections of mice expressing wildtype β-catenin (fl/fl), a D164A and a wildtype allele of β-catenin (D164A/fl), or exclusively D164A mutant β-catenin (D164A/-), as marked by arrowheads. Scale bar, 100μm.

(C) Tumor cell apoptosis was determined by immunofluorescence staining for cleaved caspase 3 (clCasp3) on tumor sections of mice expressing wildtype β-catenin (fl/fl), a D164A and a wildtype allele of β-catenin (D164A/fl), or exclusively D164A mutant β-catenin (D164A/-), as marked by arrowheads. Scale bar, 100μm.

(D) Expression of the canonical Wnt signaling targets c-Myc, Cyclin D1 and Axin2. RNA was extracted from cell lines expressing wild-type (fl/fl), wild-type and the D164A mutant form of β-catenin (D164A/-), or only D164A β-catenin treated or not with Wnt3a and analyzed for the expression of c-Myc, Cyclin D1 and Axin2 by quantitative RT-PCR.

(E) The D164A mutant form of β-catenin still binds E-cadherin. Co-immunoprecipitation (IP) using anti-β-catenin or rabbit IgG control in cell lines expressing wild-type (fl/fl) or the D164A mutant form of β-catenin (D164A/-) and subsequent immunoblotting against β-catenin, E-cadherin, and α-tubulin as loading control for the input.

(F) The D164A mutant form of β-catenin still localizes with E-cadherin at cell-cell junctions of epithelial mammary tumor cells. Immunofluorescence microscopy pictures show the localization of E-cadherin, β-catenin and E-cadherin in cell lines expressing wild-type (fl/fl) or the D164A mutant form of β-catenin (D164A/-). DAPI has been used to visualize nuclei. Scale bar, 100μm.

Suppl. Figure 6; Vafaizadeh et al.

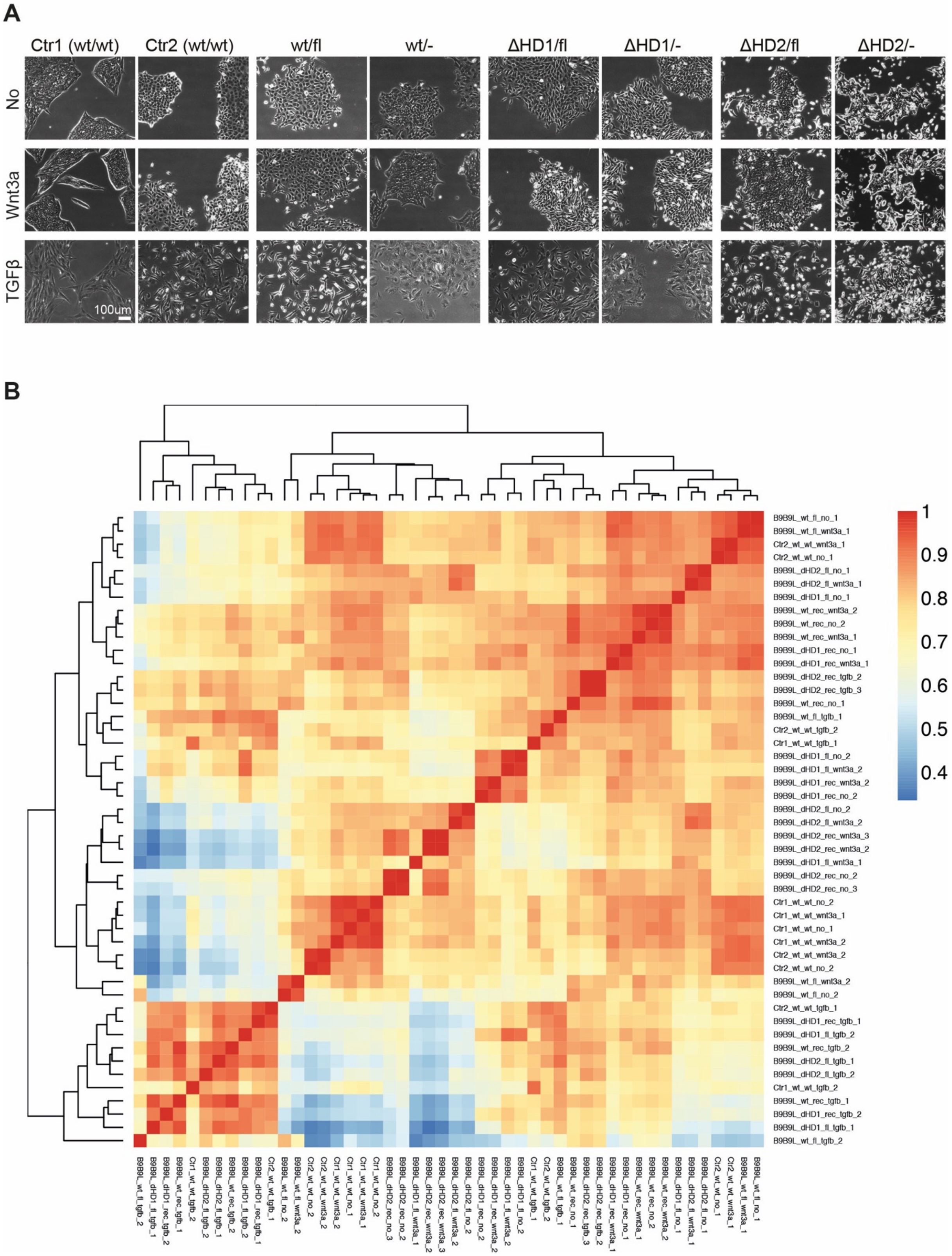

Suppl. Figure 6. Impact of disrupting the interaction of Bcl9/Bcl9L with Pygopus and β-catenin on Wnt3a and TGFβ-induced gene expression.

(A) Primary epithelial tumor cells with B9/B9L wt/wt, wt/fl, ΔHD1/fl and ΔHD2/fl genotypes were isolated from two different tumors located in thoracic mammary glands of MMTV-PyMT mice. B9/B9L wt/wt, wt/-, ΔHD1/- and ΔHD2/- transgenic cell lines were generated by infection with Cre-expressing Adenoviruses (Ad-Cre-IRES-GFP). Cells with and without Cre expressions were treated with no cytokine, Wnt3a (3 days, 100ng/ml) and TGFβ (4 days, 2ng/ml). Morphological changes and induction of EMT (lower line) are shown for one set of generated cell lines. An inhibition in EMT induction and remaining of epithelial cell islands could be only observed in cells with ΔHD2/- genotypes.

(B) Dendrogram and heatMap for Unsupervised Hierarchical Clustering of the gene expression of the various B9/B9L wildtype and mutant cell lines treated with Wnt3a (+ Wnt3a) or TGFβ (+TGFβ) and their respective untreated controls (- Wnt3a, - TGFβ). Samples do not cluster according to their genotype rather to their treatments with Wnt3a or TGFβ. Colors indicates the correlation value.

Suppl. Table I. Excel File listing the genes which are regulated by Wnt3a and are affected in their expression by the disruption of β-catenin - B9/B9L binding in the ΔHD2 mutants of B9/B9L and the D164A mutant of binding site β-catenin.

Suppl. Table II. Excel file listing the genes which are regulated by TGFβ and are affected in their expression by the disruption of β-catenin - B9/B9L binding in the ΔHD2 mutants of B9/B9L and the D164A mutant of binding site β-catenin.

Supplementary Materials and Methods

Antibodies and Reagents

Antibodies: used for immunoblotting (*WB*), immunofluorescence staining (*IF*) and immunohistochemistry (*IHC-P* or *OCT*): α-tubulin (T-9026, Sigma-Aldrich, *WB*), Alexa Fluor™ 568 Phalloidin (A12380; Invitrogen, *IF*), Axin2 (Abcam, ab32197, *WB*), β-catenin (NBP-32239, Novus Biologicals, used for *IP*), β-catenin (06-734, Cell Signaling Technology, *IF*), Bcl9 (ab37305, Abcam, *WB* and *IHC-P*), Bcl9L (Novus, NEP2-14350, *WB*), Bcl9L (PAB19408, Abnova, *IHC-P*), β-Tubulin (9F3) (2128, cell signaling, *WB*), Caspase-3 (Asp

175) (5A1E) (9664, Cell Signaling Technology, *OCT*), E-cadherin (610182, Transduction Laboratories, *WB*), E-cadherin (ECCD-2) (13-1900, Thermo Fisher Scientific, *IF*), fibronectin (F3648, Sigma-Aldrich, *WB*), GAPDH (G8795, Sigma-Aldrich, *WB*), N-Cadherin (33-3900, Zymed, *WB* and *IF*), Non-phospho (active) β-Catenin (Ser33/37/Thr41) (D13A1) (8814, Cell signaling, *WB, IF,* and *IHC-P*), phospho-Histone 3 (Ser10) (Upstate 06-570, Merck, *OCT*), PKC (MC5, P5704, Sigma-Aldrich, *WB*), P-PKC (2261, Cell signaling, *WB*), P-Stat3 (Phospho-Stat3 (Tyr705) (D3A7) (9145, Cell signaling, *WB*), Stat3 (9132, Cell signaling, *WB*), Wls (Anti-GPR177, MABS87, Merck Millipore, *WB*), Zonula occludens-1 (617300, Thermo Fisher Scientific, *IF*) and horse radish peroxidase (HRP)-conjugated secondary antibodies against mouse and rabbit (Jackson ImmunoResearch Laboratories).
Reagents: 4′,6-diamidino-2-phenylindole (DAPI, D9542, Sigma-Aldrich, *IF*), Recombinant murine Wnt3a (315-20, PeproTech) and recombinant human TGFβ1 (240-B, R&D Systems).

Histology

Tissues (mammary tumors and lungs) were fixed in 4 % paraformaldehyde overnight at 4°C followed by ethanol/xylene dehydration and subsequent embedding in paraffin. Paraffin-embedded samples were cut at 5µm thickness and subjected to Hematoxylin and Eosin (H&E) staining. To quantify lung metastasis, 9 slides per lung separated each by 50µm were stained with H&E and the number and area of lung metastases were determined under the microscope at 10x magnification.

Immunohistochemistry

Paraffin embedded tumor tissues (thoracic and abdominal mammary glands with tumors lesions) were deparaffinized and rehydrated via sequential washing with Roticlear, graded ethanol, water and PBS. Tissue sections were then subjected to high temperature–induced epitope retrieval in 10 mM citrate buffer (pH 6.0) and following permeabilization in 0.2% Triton X-100 in PBS for 20 minutes. During protein blocking, slides were treated with 5% goat serum in PBS for 1h followed by overnight incubation with specified primary antibodies at 4°C. Samples were further processed using DAKO EnVison dual link system HRP (Dako, K4065) according to manufacturer’s instructions. After staining, samples were stained with hematoxylin and then dehydrated again in graded ethanol and finally with Roticlear before mounted with cytoseal (Thermo Scientific, 8312-4). Images were captured on Leica DMI 4000 microscope.

Immunofluorescence microscopy

Tumors were fixed at 4°C in 4% paraformaldehyde for 2hrs followed by cryopreservation overnight in 20% sucrose/PBS prior to embedding in OCT-Compound freezing medium. Cryosections were cut at 7µm thickness and air-dried for 15min prior to rehydration in PBS. Tissue sections were permeabilized with 0.2% TritonX-100/PBS and blocked for 30min in 5% normal goat serum/PBS followed by incubation with the indicated primary antibodies at the appropriate dilution in blocking buffer overnight at 4°C. The next day, sections were incubated with fluorophore-coupled secondary antibodies (Alexa Fluor, Invitrogen) for 1hr at room temperature in the dark. The cell nuclei were counterstained with DAPI (D5942, Sigma-Aldrich). After staining, the coverslips were mounted in Fluorescence Mounting Medium (S302380-2, Dako) on microscope slides and imaged using a fluorescence microscope (Leica DMI 4000) at 20x magnification.

Cell culture

Established cell lines from different tumor genotypes and Py2T cells (Waldmeier et al. 2012) were cultured in Dulbecco's modified Eagle's medium (DMEM) (D5671, Sigma-Aldrich) supplemented with 10% heat-inactivated Fetal Bovine Serum (F7524, Sigma-Aldrich), 2mM L-Glutamine solution (G7513, Sigma-Aldrich), 100 U penicillin and 0.1mg/ml streptomycin (P4333, Sigma-Aldrich). All cell lines were grown at 37°C, 5% CO_2, 95% humidity. To activate Wnt/β-catenin-mediated transcription, cells were treated with 100ng/ml recombinant murine Wnt3a for the indicated time periods. For EMT experiments, cells were treated with 2ng/ml recombinant human TGFβ1 every second day for the time periods indicated.

Immunofluorescence of cultured cells

Cells were grown on glass coverslips (#1, 12mm round, Menzel–Glaser) and treated for 3 days with Wnt3a or 4 days with TGFβ. The cells were washed with PBS and then fixed with 4% paraformaldehyde/PBS for 15min at room temperature, followed by permeabilization with 0.2% TritonX-100/PBS for 5min. Subsequent blocking was performed with 3% BSA/0.0 % Triton X-100/PBS for 1hr. Primary antibodies were then added in the appropriate dilution in 3% BSA/0.2% PBS-T for 2hrs at room temperature. Incubation with fluorophore-coupled secondary antibodies (Alexa Fluor, Invitrogen) was performed for 1hr at room temperature in the dark. Cell nuclei were counterstained with DAPI (D5942, Sigma-Aldrich). After staining, coverslips were mounted in Fluorescence Mounting Medium (S302380-2, Dako) on microscope slides and imaged using a fluorescence microscope (Leica DMI 4000) at 20x

magnification.

Immunoblotting

Cells were lysed in RIPA buffer (R0278, Sigma-Aldrich) supplemented with 1mM DTT, mM NaF, 2 mM sodium orthovanadate and 1X protease inhibitor cocktail (Sigma-Aldrich) for 30 min on ice followed by scraping into tubes and centrifugation for 10min at 10,000 rpm at 4°C. The supernatant was saved and the protein concentration was determined using a Bio-Rad Bradford assay according to the manufacturer's instructions. Equal amounts were prepared, diluted in loading buffer (10% glycerol, 2% SDS, 65mM Tris, 0.01mg/ml Bromphenolblue, 1% β-mercaptoethanol) and loaded onto a SDS polyacrylamide gel. Proteins were then transferred onto nitrocellulose membranes (10600002, Sigma-Aldrich) by wet transfer for 2hrs at constant current (0.33 A). Following blocking for 1hr in 5% milk prepared in TBS/0.05% Tween 20, membranes were incubated with primary antibodies overnight at 4°C. The next day, membranes were washed and incubated with HRP-conjugated secondary antibodies (Jackson Immunoresearch Laboratories and donkey anti goat IgG-HRP, sc-2020, Santa Cruz) for 1hr at room temperature and developed with Immobilon Western Chemiluminescent HRP Substrate (WBKLS0500, Millipore) and quantified using a Fusion Fx7 chemiluminescence reader.

Co-immunoprecipitation

β-catenin$^{fl/fl}$ or β-catenin$^{D164A/-}$ cells were grown in 15cm plates, washed with cold PBS, scraped off in 300µl IP lysis buffer (20mM Tris pH 7.5, 10mM NaCl, 10% glycerol, 1% NP-40, 2mM EDTA, 1X PIC (protein inhibitor cocktail)) and collected in an Eppendorf tube. Incubation was performed at 4°C rotating for 20min. Subsequently samples were centrifuged at 4°C (10min, 16,000g), and supernatants were transferred to a new tube. Protein concentrations were measured using Bio-Rad Bradford solution according to the manufacturer's instructions. An input sample was taken and frozen. Per IP, 5µg antibody or IgG as a control were diluted in 200µl PBS/0.02% Tween-20 (PBS-T). Per IP 40µl DynaMagnetic Beads were prepared by washing 2 times with 500µl PBS-T. Beads were collected using a magnetic stand, antibody dilution was added and rotated for 15min at room temperature. The liquid was aspirated on the magnetic stand and the lysate (1mg) was added and incubated overnight at 4 °C while rotating. The next day, beads were washed three times with 500µl IP wash buffer (15mM Tris-HCl pH 7.8, 100mM NaCl, 1X PIC) and then diluted in 40 µl 1X SDS-PAGE sample buffer and boiled for 5min. Empty beads were collected and the supernatant was loaded and analyzed by SDS-PAGE.

RNA-isolation and RT-qPCR

RNA was isolated by Trizol/chlorophorm extraction and reverse-transcribed to cDNA, and gene expression was determined by quantitative real-time PCR using PowerUp SYBR Green (ThermoFisher Scientific) with the following primers:

Bcl9 (Fw: CAATCAATGCATCCCAGTAACC, Rev: AGATGGGGACATCACTGTAGG)
Bcl9L (Fw: ACTCCATCCCTCGATTCAGA, Rev: CCCACTGTACGGCTGCTT)
RPL19 (Fw: CTCGTTGCCGGAAAAACA, Rev: TCATCCAGGTCACCTTCTCA, used to normalize gene expression between samples)
N-cadherin (Fw: CAATGACGTCCACCCTGTTCT,
Rev: CTGCCATGACTTTCTACGGAGA)
E-cadherin (Fw: CGACCCTGCCTCTGAATCC, Rev: TACACGCTGGGAAACATGAGC),
Itga5 (Fw: CACCACCATTCAATTTGACAGCA, Rev: GCTCCTCTCCCTTGGCACTGTA)
Wnt5a (Fw: TGAAGAAGCCCATTGGAATA, Rev: AGAAAAACGTGGCCAAAGC)
Wnt5b (Fw: AGCACCGTGGACAACACAT, Rev: AAGGCAGTCTCTCGGCTACC)
Wnt9a (Fw: ATGCTGGATGGGTCCCTTCT, Rev: ACTGCCTGTTAGCCCGAAGTA)
Wnt11 (Fw: CAGGATCCCAAGCCAATAAA, Rev: GTAGCGGGTCTTGAGGTCAG)
Wls (Fw: CCCAGCCATGAGCAAAGT, Rev: GCATGAGGAACTTGAACCTGA)
Fzd1 (Fw: ATCTGGTCCGGCAAGACA, Rev: GCTGTTGGTAAGCCTCGTGT)
Fzd2 (Fw: CCGACGGCTCTATGTTCTTC, Rev: TAGCAGCCGGACAGAAAGAT)
Lef1 (Fw: TCCTGAAATCCCCACCTTCT, Rev: TGGGATAAACAGGCTGACCT)
c-Myc (Fw: GAGACACCGCCCACCACCAGC, Rev: GGCACCTCTTGAGGACCAGTGG)
Cyclin D1(Fw: CCAGAGTCATCAAGTGTGACC, Rev: CACGTCGGTGGGCGTGCAGG)
Axin2 (Fw: GGGGGAAAACACAGCTTACA, Rev: TTGACTGGGTCGCTTCTCTT)
